# Integrated omics-based pathway analyses uncover CYP epoxygenase-associated networks as theranostic targets for metastatic triple negative breast cancer

**DOI:** 10.1186/s13046-019-1187-y

**Published:** 2019-05-09

**Authors:** Maria Karmella Apaya, Jeng-Yuan Shiau, Guo-Shiou Liao, Yu-Jen Liang, Chia-Wei Chen, Hsin-Chou Yang, Chi-Hong Chu, Jyh-Cherng Yu, Lie-Fen Shyur

**Affiliations:** 1 Molecular and Biological Agricultural Sciences Program, Taiwan International Graduate Program, Academia Sinica, Taipei 115, Taiwan and National Chung Hsing University, Taichung, 402 Taiwan; 2 Agricultural Biotechnology Research Center, Academia Sinica, Taipei, 115 Taiwan; 3 Graduate Institute of Biotechnology, National Chung Hsing University, Taichung, 402 Taiwan; 4 Tri-Service General Hospital, National Defense Medical Center, Taipei, 114 Taiwan; 5 Institute of Statistical Science, Academia Sinica, Taipei, 115 Taiwan; 6 Biotechnology Center, National Chung Hsing University, Taichung, 402 Taiwan; 7 PhD Program in Translational Medicine, College of Medicine, Kaohsiung Medical University, Kaohsiung, 807 Taiwan

**Keywords:** CYP450 epoxygenase, Epoxyeicosatrienoic acid, Metastasis, Oxylipin metabolome, Triple negative breast cancer

## Abstract

**Background:**

Current prognostic tools and targeted therapeutic approaches have limited value for metastatic triple negative breast cancer (TNBC). Building upon current knowledge, we hypothesized that epoxyeicosatrienoic acids (EETs) and related CYP450 epoxygenases may have differential roles in breast cancer signaling, and better understanding of which may uncover potential directions for molecular stratification and personalized therapy for TNBC patients.

**Methods:**

We analyzed the oxylipin metabolome of paired tumors and adjacent normal mammary tissues from patients with pathologically confirmed breast cancer (*N* = 62). We used multivariate statistical analysis to identify important metabolite contributors and to determine the predictive power of tumor tissue metabolite clustering. In vitro functional assays using a panel of breast cancer cell lines were carried out to further confirm the crucial roles of endogenous and exogenous EETs in the metastasis transformation of TNBC cells. Deregulation of associated downstream signaling networks associated with EETs/CYPs was established using transcriptomics datasets from The Cancer Genome Atlas (TCGA) and Molecular Taxonomy of Breast Cancer International Consortium (METABRIC). Comparative TNBC proteomics using the same tissue specimens subjected to oxylipin metabolomics analysis was used as validation set.

**Results:**

Metabolite-by-metabolite comparison, tumor immunoreactivity, and gene expression analyses showed that CYP epoxygenases and arachidonic acid-epoxygenation products, EET metabolites, are strongly associated with TNBC metastasis. Notably, all the 4 EET isomers (5,6-, 8,9-, 11,12-, and 14,15-EET) was observed to profoundly drive the metastasis transformation of mesenchymal-like TNBC cells among the TNBC (basal- and mesenchymal-like), HER2-overexpressing and luminal breast cancer cell lines examined. Our pathway analysis revealed that, in hormone-positive breast cancer subtype, CYP epoxygenase overexpression is more related to immune cell-associated signaling, while EET-mediated Myc, Ras, MAPK, EGFR, HIF-1α, and NOD1/2 signaling are the molecular vulnerabilities of metastatic CYP epoxygenase-overexpressing TNBC tumors.

**Conclusions:**

This study suggests that categorizing breast tumors according to their EET metabolite ratio classifiers and CYP epoxygenase profiles may be useful for prognostic and therapeutic assessment. Modulation of CYP epoxygenase and EET-mediated signaling networks may offer an effective approach for personalized treatment of breast cancer, and may be an effective intervention option for metastatic TNBC patients.

**Electronic supplementary material:**

The online version of this article (10.1186/s13046-019-1187-y) contains supplementary material, which is available to authorized users.

## Background

Molecular subtyping and immunohistopathological (IHC) examination of estrogen receptor (ER), progesterone receptor (PR) and human epidermal growth factor receptor (HER2) status serve as frontline guides for clinical decision-making in breast cancer (BC) therapy [1, 2]. Current therapeutic approaches, however, are still ineffective for triple negative breast cancer (TNBC, ER−/PR−/HER2−) patients, which account for approximately 15% of all invasive mammary tumors [3, 4]. Currently, IHC analysis and gene expression profiling are the methods used for sub-classification of “TNBC” tumors, which may include basal-like 1, basal-like 2, immunomodulatory, mesenchymal, mesenchymal-stem-like, and luminal androgen receptor-positive clusters [5–8]. However, this sub-classification scheme is ambiguous and still has limited translational applications [8, 9]. Although systemic chemotherapy may attenuate the aggressive nature of the disease, most TNBC patients have a disproportionally high tendency to develop rapid onset visceral and distal metastasis, recurrence, and decreased survival rate [6–8]. Better understanding of molecular signatures for TNBC is thus needed to identify prognostic markers for clinical outcome prediction and to develop suitable personalized medicine for TNBC patients.

In recent years, the development of analytical techniques for genomics, proteomics and metabolomics analyses has provided a more global insight into the molecular biology of TNBC [9]. For example, gene expression profiling helps stratify molecular features of TNBC tumors [10–12], methylome sequencing data has highlighted the prognostic value of epigenetic changes [13] and global metabolomics and proteomics analyses correlate decreased citrate, increased sarcosine and 2-hydroxyglutarate levels, and differentiated long-chain fatty acid metabolism-related proteins with the different BC subtypes [14, 15]. However, information derived from individual omics data types may be constrained to the discovery of less inclusive molecular signatures with limited translational applications. Integrating biological information derived from cohorts of multiple level high resolution molecular analysis, e.g., The Cancer Genome Atlas (TCGA), with multi-omics, cross-platform data comparison may ultimately lead to refinement of existing diagnostic classification and therapeutic options for TNBC.

Recent lipidomics findings show that phospholipids and membrane-derived mediators are the main contributors to the aggressive and metastatic phenotype of ER or PR negative tumors [16]. These lipid autocoids influence inter- and intracellular metabolic and inflammatory signaling, which are two important cancer hallmarks [17, 18]. Among these lipid products, eicosanoids, a group of metabolites derived from oxidative transformation of arachidonic acid (AA) by cyclooxygenase (COX), lipoxygenase (LOX) or cytochrome P450 (CYP) enzymes may have a major impact on malignant cell transformation and TNBC metastasis progression [19]. While the cancer-associated eicosanoids derived from COX and LOX, e.g., prostaglandin E_2_ (PGE_2_) and hydroxyeicosatetraenoic acids (HETEs), are well-established, the roles of CYP-derived mediators are more enigmatic [20, 21]. Aside from the xenobiotic metabolizing CYPs primarily found in the liver, which are important in drug metabolism and resistance, the pro-metastatic, angiogenic and anti-apoptotic functions of extrahepatic CYPs (the CYP2J and CYP2C families) are of interest in cancer research [22–25].

CYP2J/2C catalyzes conversion of AA to 4 regioisomeric epoxyeicosatrienoic acids, viz., 5,6-EET, 8,9-EET, 11,12-EET and 14,15-EET [21]. Several studies utilizing in vitro and in vivo models have highlighted the relationship between specific EET (e.g., 14,15-EET) signaling and breast cancer progression [23, 26–28]. Expression of CYP2C8/9 and CYP2J2, and/or high level of 14,15-EET detected in patients were found to be positively correlated with aggressiveness of human BC [29], or that might be involved in breast cancer cell epithelial-mesenchymal transition and cisplatin resistance [30]. The biological significance of these findings and their translational implications, however, are still elusive because information on the global and comprehensive effects of all EET metabolites, alone or in combination, on breast cancer metastasis have not been closely examined. We hypothesized that isomers of this endogenous EET metabolite class are key players in regulating the multi-layered network signaling in breast cancer. In particular, we are interested in exploring the roles of EETs in hormone-independent signaling processes in TNBC.

In this study, we examined the global oxylipin metabolite profiles of breast cancer tumors and show the importance of EET metabolites in relation to hormone receptor subtype, metastasis status and prospective clinical outcomes. We used information derived from integrative genomics, proteomics and metabolomics coupled with multi-source genetic, cellular and tissue analyses from public cohorts, i.e., The Cancer Genome Atlas (TCGA) and the Molecular Taxonomy of Breast Cancer International Consortium (METABRIC) data sets, or human BC cells or patient specimens. Our findings expose a new set of vulnerabilities and associated biomarkers within the TNBC lipid metabolite-protein-gene network and provide a clinically-relevant roadmap for the development of personalized intervention strategies for TNBC patients.

## Methods

### Reagents

All chemicals and solvents were used as purchased. Oxylipin standards were purchased from Cayman Chemicals (Ann Arbor, MI). Antibodies used for western blotting and immunohistochemical staining were: FAK, p-FAK, Src, p-Src, EGFR, and p-EGFR obtained from Cell Signaling Technology (Beverly, MA); and CYP2B, CYP2C9/19, CYP2J, CYP3A, and flotillin-1 purchased from Santa Cruz (Santa Cruz, CA).

### Cell cultures

Human-derived TNBC (MBA-MB-231, Hs 578 T, MDA-MB-468, HCC1937 and BT-20), ER+/PR+/HER2- (MCF7, ZR75–1 and BT483), and HER2-overexpressing (SKBR3, MDA-MB-361 and BT474) cell lines and normal mammary epithelial cells (MCF10A) were all originally obtained from the American Type Culture Collection (Manassas, VA). Hs 578 T and MDA-MB-468 were gifts from Dr. Ruey-Hwa Chen of the Institute of Biological Chemistry, Academia Sinica, Taiwan. HCC1937, BT-20, ZR75-1, BT483, MDA-MB-361 and BT474 are obtained from Dr. Wen-Hua Lee, Genomics Research Center, Academia Sinica, Taiwan.

### Functional experiments in human mammary cancer cell lines

MTT assay was performed to assess cell viability. Expression levels of proteins of interest were analyzed through western blotting. Transwell migration and invasion experiments were performed to measure the metastatic potential of cells. Migration experiment was carried out as follows: cells were placed into the upper chamber (5 × 10^4^ cells per insert) with DMEM culture medium containing 0.1% FBS. Ten percent FBS was added to the lower well (6.5 mm diameter, 8 mm pore size; Costar, Cambridge, MA) as a chemoattractant. Cells were then treated with vehicle (DMSO, 0.5%) or inhibitors. After 24 h incubation, non-migrating cells were scraped from the upper surface of the membrane using a cotton swab. Cells remaining on the underside were fixed and stained with DAPI solution (1 mg/ml) and counted at 20x original magnification by inverted fluorescence microscopy. For invasion experiments, 8 mm filters were pre-coated with Matrigel (30 mg/filter) and incubated at 37 °C for 2 h, prior to following the protocol of migration assay. Three-dimensional culture models of normal and malignant mammary cells were established based on previously published protocols [31]. A Zeiss LSM 780 plus Elyra confocal microscope was used to visualize and capture the stained 3D acini.

### Gene knockdown using the shRNA technique

The shRNA clones were purchased from the National RNAi Core Facility Academia Sinica (Taiwan). shRNA sequences and target genes used are: CYP2C19: 5′-CGGCCCTGTGTTCACTCTGTATTTCTCGAGAAATACAGAGTGAACACAGGGTTTTTG-3′; CYP4A2: 5′-CCGGCTTGCTCTCCCAGGATCAATTCTCGAGAATTGATCCTGGGAGAGCAAGTTTTTG-3′; LacZ (control): 5′-CCGGCCGTCATAGCGATAACGAGTTCTCGAGAACTCGTTATCGCTATGACGGTTTTTG-3′. Gene and protein expression levels of target genes were analyzed by qRT-PCR and western blotting.

### Real-time quantitative PCR

For real time PCR, total RNA was isolated using RNeasy Mini Kit (Quiagen). cDNA was generated by reverse transcription of RNA aliquots using the Takara PrimeScript RT Reagent Kit (Takara) according to the manufacturer’s instruction. The resulting cDNA was used for real-time PCR with SYBR® Premix Ex Taq™ Kit (Takara) in a StepOne Real-Time PCR Detection System (Life Technologies). All expression data were normalized to GADPH-encoding transcript levels. Primers used for real-time PCR are as follows: CYP2C19: (F:5′-CTTCTGTCCCGCCCTTCTATC-3′) (R:5′-GATAGTGAAATTTGGACCAGAGGA-3′); CYP2J2: (F:5′-GAAGGGCTTAGAGGAACGCA-3′) (R:5′- AGCGTTCTCCGAAGGTGATG-3′); sEH: (F:5′-TGCCCAGAGGACTTCTGAATG-3′) (R:5′-TTGGGGAGGCAGACTTTAGC-3′); GADPH: (F:5′-AGGGCTGCTTTTAACTCTGGT-3′) (R: 5′-CCCCACTTGATTTTGGAGGGA-3′).

### Study population

Four data sets were used in this study: a data set from our own group, used for bioactive lipid mediator-targeted metabolomics and proteomics analysis; an mRNA microarray data set from TCGA breast cancer specimens and a gene expression data set from the Molecular Taxonomy of Breast Cancer International Consortium (METABRIC) (downloaded from cBioportal), used for pathway deregulation analysis. We selected systemically untreated patients from the following GEO data sets (GSE19615, GSE21653, GSE2603, GSE31519, GSE45255, GSE17907, GSE20271, GSE37946, GSE19615, GSE2603) from the online tool kmplotter [32] to generate survival plots correlated with expression of EET metabolizing enzymes. Our metabolomics cohort is composed of 62 paired tissue specimens derived from breast tumors and normal mammary tissues obtained during the same surgical procedure in patients. The specimens with mammary lesions were diagnosed at the National Defense Medical Center (NDMC), Taipei, Taiwan. This study was approved by the institutional review boards of the NDMC (IRB number: TSGHIRB-099-05-058). All participants signed an informed consent. Additionally, we downloaded and processed TCGA breast cancer mRNA expression data from 2007 tumors and relevant adjacent normal tissue controls from the TCGA data portal (https://www.cancer.gov/about-nci/organization/ccg/research/structural-genomics/tcga/?redirect=true) using the R package “TCGA-Assembler.” Normalized gene expression data from 1981 patients from the METABRIC cohort were also analyzed. Data from the TCGA and cBioportal were accessed and retrieved in November 2018.

### Data set configurations for model training, validation, and testing

We used 53 of the specimens for metabolomics analysis as discovery and the remaining 9 as a validation data set. TCGA mRNA expression data (Agilent microarray) was used as a training set for pathway deregulation analysis while METABRIC was used for validation and testing. There was no sample overlap between the discovery/training and validation/test sets. Proteomics data derived from 8 TNBC samples used in the metabolomics study were used to test applicability of TNBC subtyping from PDS scores.

### Collection, storage and handling of mammary tissue specimens

Tissue specimens from patients were flash frozen in liquid N_2_ and stored at -80 °C for long-term storage. Noncancerous mammary tissues were collected at least 5 cm away from the margins of tumors and were defined as normal breast tissues present without ductal carcinoma in situ (DCIS), atypical hyperplasia or benign breast disease by pathological confirmation. Normal tissue samples were selected to contain 80–100% normal ductal or peri-ductal tissue and only 0–20% adipose tissue, to ensure comparability with the tumor samples. Clinicopathological information for the tumors including treatment modalities or expression of hormone receptors and HER2 was obtained from the review of medical records and pathology reports. Tumors with ≥1% nuclear-stained cells by IHC were considered positive for estrogen receptor (ER) and progesterone receptor according to the American Society of Clinical Oncology/College of American Pathologists (ASCO/CAP) guidelines. HER2, ER and PR immunostaining were scored as 0, 1+, 2+, or 3+ according to ASCO/CAP guidelines.

### Oxylipin metabolome analysis

Levels of bioactive lipid mediators in quiescent cells and in frozen tumor and normal mammary samples were determined using an optimized an ultra-performance liquid chromatography-mass spectrometry (UPLC-MS/MS) protocol with minor modifications [33]. Only tissue samples containing ≤20% adipose tissue upon histopathological quality control were included in the metabolomics analysis. Liquid-liquid extraction on 100–200 mg tissue samples was conducted using chloroform:methanol (2:1), vortexed for 2 min, incubated for 30 min at 4 °C and centrifuged at 13000×g for 10 min. Lipid extracts were analyzed on UPLC-MS/MS system (ACQUITY UPLC, Waters) coupled with a TSQ Quantum Access Max (Thermo Fisher Scientific, USA) triple quadrupole mass spectrometer, operated in a negative MRM mode and fitted with an ACQUITY UPLC HSS BEH column (particle size 1.8 μm, 2.1 × 100 mm, Waters) at 400 μl/min flow rate using a 25 min gradient for analysis. Mobile phase A consisted of 0.1% NH_4_OH in water and mobile phase B consisted of 0.1% NH_4_OH in MeOH. The chromatogram acquisition, detection of mass spectral peaks and waveform processing were performed using ThermoXcalibur 2.1 SP1 software (Thermo Scientific). The calibration curve and quantification were performed using LCQuan 2.6.1 software (Thermo Scientific). The peak area of each quantified ion was calculated and normalized against the peak area of the corresponding internal standards. Coefficient of variation (CV), < 15% for five technical replicates and < 30% for each biological sample, respectively, was used to assess data inclusion.

### Mass spectrometry (MS)-based quantitative proteomic analysis of clinical TNBC specimens

Total protein extracts from 8 clinical TNBC tumor and adjacent normal tissue specimens were obtained using dual lysis buffer system as previously reported [34]. Protein extracts from combining both lysis buffers were precipitated by adding 6 volumes of cold acetone containing 10% trichloroacetic acid (TCA) at − 20 °C overnight, and then pelleted through centrifugation at 13,000×*g* for 15 min at 4 °C and air-dried. The protein pellet was dissolved with 8 M urea in 50 mM Tris buffer (pH 8.5), and the protein concentrations were measured by Pierce 660 nm protein assay (Thermo Scientific, Rockford, USA). The protein digestion, isobaric tags for relative and absolute quantification (iTRAQ) labeling, proteolytic peptide fractionation and LC-MS/MS analysis, and protein identification or quantification were carried out according to the method previously described. The 8 TNBC tumor and adjacent normal tissue specimens in this study were divided into two groups, TNBC-1 to 4 and TNBC-5 to 8, and each group was labeled with 8-plex iTRAQ reagent (AB SCIEX, Foster City, CA). Peptide and protein identification was performed using the Proteome Discoverer software (v.1.4.1.14., Thermo Fisher Scientific) with SEQUEST and MASCOT search algorithms (Matrix Science) against a Swiss-Prot human protein database of Human uniprot 148,986 entries. The parameters for database searches were set as follows: full trypsin digestion with 2 maximum missed cleavage sites, precursor mass tolerance of 10 ppm, fragment mass tolerance of 0.02 Da, dynamic modifications of oxidation at methionine (M) residues, and static modifications of carbamidomethylation at cysteine (C) residues, iTRAQ 8-plex at lysine residues and N-terminal proteolytic peptides. The identified peptides were validated using Percolator algorithm against the decoy database search which rescored peptide spectrum matches (PSM) by q-values and posterior error probabilities. All the peptides were filtered with the identified protein having a minimum of two unique peptides. For normalization of iTRAQ-labeled peptide ratios, Proteome Discoverer software (v.1.4.1.14., Thermo Fisher Scientific) contains the normalization factor to correct experimental bias. For quantitative analysis, the relative abundance of each protein present in two biological replicates was calculated based on the iTRAQ reporter ion ratios of 115/114 and 116/114 found at the peptide level.

### Immunohistochemical staining

IHC was performed using whole sections of formalin-fixed, paraffin-embedded tissue block (N-Histofine® Simple Stain AP, Nichirei Biosciences, Tokyo, Japan). Color developing was done using 3,3′-diaminobenzidine and slides were counterstained with hematoxylin. The primary antibody incubation step was omitted in the negative control. Images were taken using Zeiss Axioimager Z1 and processed using Carl Zeiss ZEN software 11. Automated scoring was conducted using IHC Profiler; an Image J plugin was used for quantitative analysis of immunoreactivity of tumor tissues against CYP2J2, CYP2C19, CYP3A4 and sEH antibodies. Percentile score of negative/weak positive, positive, and strongly positive DAB-stained cytoplasmic zones were calculated using a pixel-by-pixel scoring analysis along the whole image profile [35].

### In silico association of related enzymes to receptor status and survival

Survival analysis for both the TCGA and METABRIC datasets was performed using the R package “survival.” Patient follow-up time was limited to 5 years, and only breast cancer-related deaths were counted. The probability of overall survival of systematically untreated patients based on CYP450 epoxygenase expressions for validation using independent cohorts was calculated using the Kaplan-Meier plotter database (http://kmplot.com/analysis/index.php?p=service&cancer=breast) accessed in January 2018. Affymetrix probe set IDs selected for the evaluation of CYP2B6, CYP2C18, EPHX3, CYP4A2, CYP2J and CYP3A4 were 219825_at, 215103_at, 220013_at, 206514_s_at, 205073_at, 205998_x_at. Hazard ratio with 95% confidence interval and log-rank *P* value were calculated and reported as displayed on the webpage. All other parameters were set to default.

### Network association analysis

To correlate metabolomics data with protein and gene network relationships in TNBC, we first performed a single point statistical test using Wilcoxon signed-rank test on metabolites of interest and corresponding CYP enzymes (WSRT, pFDR < 0.05). Using cBioportal, we used gene enrichment analysis of TCGA and METABRIC datasets to identify top enriched and co-expressed genes. The list of CYP epoxygenase amplification-associated genes was generated by performing differential gene-expression analysis using a standard linear modeling procedure. Generated *P* values were corrected for multiple testing by controlling the false discovery rate (FDR) across genes using the Benjamini and Hochberg correction and by adopting the nested F correction across contrasts. Genes were grouped according to gene ontology (GO) molecular functions and biological processes to construct a comprehensive cancer-associated sub-network. Pathway and network enrichment analysis (with Bonferroni corrections) between metabolite, biosynthetic enzyme and related genes and biological processes were then performed using Metscape 3.1 App for Cytoscape 3.5.

### Assembly of pathway associated gene sets and pathway deregulation analysis

Gene sets were imported from three pathway databases, KEGG, BioCarta and Reactome, downloaded from the MSigDB collections. The oncogenic signature and cancer hallmark signature gene set collections, also from MsigDB were included. A total of 186 KEGG, 217 BioCarta, 674 Reactome, 50 hallmark and 189 oncogenic signature pathways were analyzed. Gene identity was established according to their official gene symbols.

We used the R package “Pathifier” to perform pathway-based gene set analysis. The information derived from Pathifier was related to metabolite pathways and molecular functions, e.g., metastasis and proliferation, as well as characteristics of individual breast tumors, receptor subtype and status of CYP epoxygenase expression. This algorithm transforms gene-level information from curated data sets to pathway-level deregulation scores [36].

### Functional enrichment analysis

WEB-based GEne SeT AnaLysis Toolkit [37] or STRING [38], a functional protein association tool were used to analyze and visualize protein networks. For the functional enrichment analysis, we relied on the overrepresentation enrichment analysis (ORA) and on the Network Topology-based Analysis functionalities. The *P* values for this analysis were adjusted with the Holm-Bonferroni method and the false discovery rates were reported. The gene list enrichment analysis was conducted according to all known human genes or against RNAseq data curated from TCGA–BRCA, applying a correction for multiple testing.

### Data processing and statistical analysis of metabolomics data

Multivariate data analysis was carried out using SIMCA-P 11.0 software (Umetrics, SE). Metabolites included in the statistical analyses were those which were consistently detected in at least 80% of samples. All known artifact peaks, such as internal standards, column bleed, plasticizers, or reagent peaks, were excluded. Tissue type and receptor statuses were used as grouping variables for visualization of data. Fisher’s linear discriminant analysis (LDA) and linear support vector machines (SVM) were used to construct predictive signatures. The robustness of the classifier models were validated in a built-in iterative 7-fold leave-one-out approach. The resulting distributions of average prediction rates were visualized as tables. Univariate analyses and Euclidian hierarchical classification were carried out without replacement of missing data.

### Data and code availability

Sample information and mRNA datasets for both the TCGA and METABRIC breast cancer specimens were retrieved from https://portal.gdc.cancer.gov/ and http://www.cbioportal.org/. Survival data for independent datasets were downloaded from http://kmplot.com/analysis. Codes used in this study were adopted from https://github.com/compgenome365/TCGA-Assembler-2 for TCGA Assembler, and https://bioconductor.org/packages/release/bioc/html/pathifier.html for Pathifier analysis. http://www.webgestalt.org/ was accessed as an online tool for the identification of subtype-specific pathways and over representation analysis (ORA) and network topology-based analysis (NTA A summary of publicly available information and websites used in this study is presented in Additional file 1: Table S1.

## Results

### Oxylipin metabolome profiling differentiates normal and tumor tissues and stratifies BC subtypes based on receptor status

**Fig. 1:**
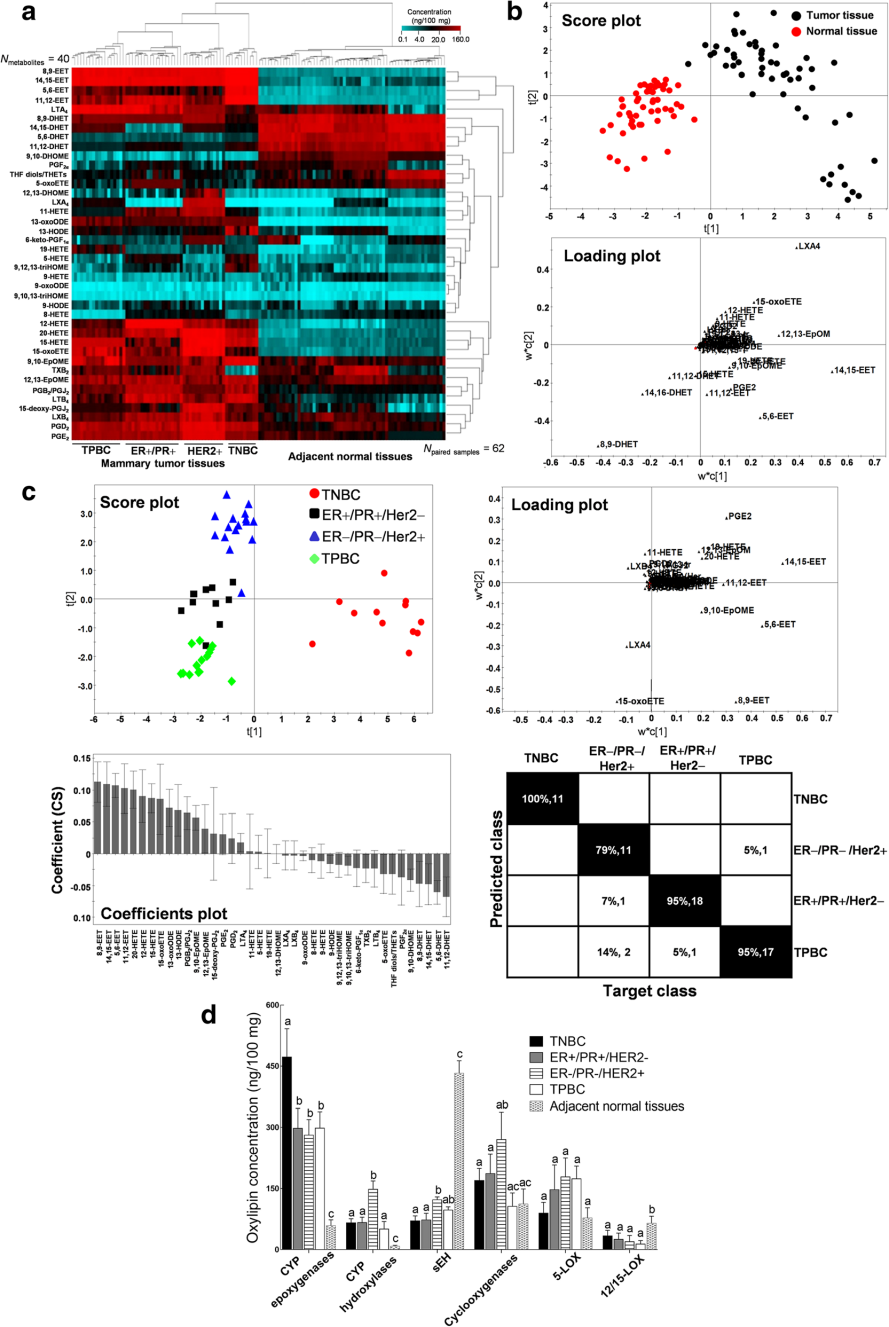
Comparative oxylipin metabolite profiling differentiates breast tumors according to receptor subtypes. **a** Heat map showing hierarchical clustering of paired tumor and normal mammary tissue specimens (*N* = 62) and oxylipin metabolites (*N* = 40) reveals that both tumor and normal samples are grouped into two distinct groups based on the Euclidian correlations. **b** Cross validated PLS-DA score and loading plots show the clustering of tumor and normal mammary tissues. Each biological replicate is represented by a single point. **c** Cross validated PLS-DA analysis comparing mammary tumors by hormone receptor status highlights the contribution of each oxylipin metabolite to the clustering in the score, loading and coefficients profile. Prediction matrix shows the classification results based on Fisher’s probability (91.9% correct assignment, *P* = 4.5E-23). **d** Total concentration of AA and LA-derived oxylipin metabolites from the CYP epoxygenases, CYP hydroxylases, sEH, cyclooxygenases, 5-LOX and 12/15-LOX catalytic pathways were quantified in the tumor and normal adjacent tissue specimens (*N* = 62). All analyses include 4 technical replicates per biological sample. Error bars indicate mean ± SEM. Significantly different values (*P* = 0.05, ANOVA) are denoted by letters

An LC-MS/MS-based targeted metabolomics platform that was developed in-house, covering eicosanoids and other oxidized fatty acid metabolites, was utilized to investigate the oxylipin dynamics in paired normal mammary and tumor tissues in a cohort of patients with pathologically confirmed breast cancer (*N* = 62). Baseline clinico-pathologic data are summarized in Additional file 1: Table S2. Identity and absolute quantification of individual oxylipin metabolites (ng/100 mg tissue) are presented in Additional file 1: Table S3. Hierarchical analysis using Euclidian distance parameters represented as heatmap clusters highlight metabolite classifiers for adjacent normal mammary and tumor tissues (Fig. 1a). CYP-catalyzed epoxygenation products, EETs and epoxyoctadecenoic acids (EpOMEs), as well as the pro-inflammatory or cancer-associated prostaglandin metabolites, PGE_2_ and PGD_2_, HETEs and leukotrienes (LTs) are elevated in all tumors compared with adjacent normal mammary tissues. Notably, EET hydroxylation products, dihydroxyeicosatrienoic acids (DHETs), were at higher concentrations in normal tissues. Among the four different breast tumor types, CYP epoxygenase products, EETs, were elevated the most in TNBC while several lipoxygenase-derived AA metabolites including lipoxins (LXs), leukotrienes (LTs), 5-HETE and 15-HETE were significantly elevated in ER+/PR+/HER2- or TPBC tumor tissues. CYP hydroxylase and cyclooxygenase products, e.g., 20-HETE and prostaglandin derivatives, were most significantly elevated in ER−/PR−/HER2+ tumors.

Clustering of tissue samples and signature metabolite contributors in the cross-validated score and loading plots were generated using supervised multivariate statistical analysis partial least squares-discriminant analysis (PLS-DA) (Fig. 1b). These results establish the association between deregulated oxylipin metabolism and mammary malignancy in paired tissue samples. We further showed that oxylipin metabolites differentiate breast cancer tumor subtypes (Fig. 1c). Remarkably, TNBC tumors were clustered separately from other groups in the score plot. As demonstrated in the loading and coefficients plots, tumor tissue classification is mainly contributed by the elevated 5,6-EET, 8,9-EET, 11,12-EET and 14,15-EET concentrations in TNBC tumors. This method correctly classified 57 out of 62 tumors based on receptor subtype, leading to a prediction accuracy of 91.9%.

The summation of oxylipin concentrations grouped by the specific catalytic enzyme class is presented in Fig. 1d. TNBC tumors are characterized by high levels of CYP epoxygenase metabolites, a unique and significant feature, compared to the other tumor subtypes. CYP hydroxylase and sEH metabolites are most significantly elevated in ER−/PR−/HER2+ tumors, while there is no statistical difference among the metabolites derived from the six catalytic enzymes in ER+/PR+/HER2− tumors and TPBC tumors. Concentration levels of CYP-derived hydroxyl and epoxy metabolites were significantly lower and sEH and 12/15-LOX metabolites were significantly higher in adjacent normal tissues than the tumor tissues.

### EET metabolite levels corroborate with gene expression and immunoreactivity of CYP epoxygenases in TNBC tissues

**Fig. 2:**
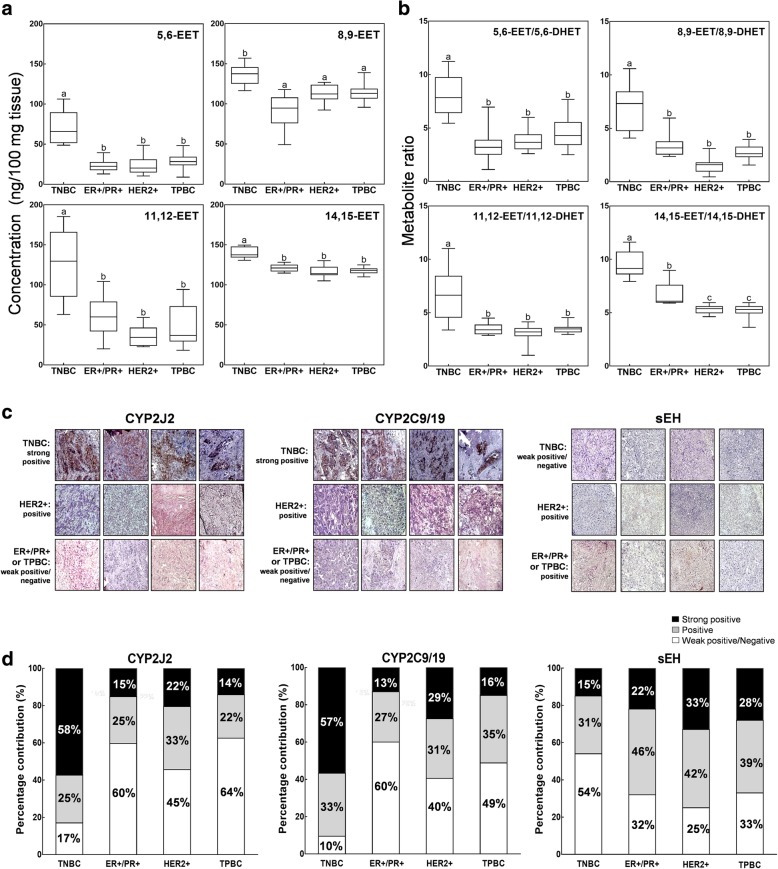
Comparative analysis of EET metabolite levels or CYP epoxygenase expressions may serve as potential bio- and histologic markers for TNBC. Box plots showing the concentration of the **a** EET isomers and the **b** EET/DHET ratios detected and quantified in breast cancer tumors (*N* = 62) classified according to hormone receptor status: TNBC (ER−/PR−/HER2−), HER2− (ER+/PR+/HER2-), HER2+ (ER−/PR−/HER2+) and TPBC (ER+/PR+/HER2+). All analyses include 4 technical replicates per biological sample. Error bars indicate mean ± SEM. Significantly different values (*P* = 0.05, ANOVA) are denoted by letters. **c** Representative strong positive, positive and weak positive/negative immunostaining results of IHC analyses for tumor sections (*N* = 55) obtained against CYP2J, CYP2C or sEH antibodies. DAB-staining was used to visualize immunoreactive regions and cellular nuclei (blue) were counterstained with hematoxylin. Images were taken at 40x magnification. **d** Quantitative scoring analyses of the immunostained tissues using IHC profiler shows the percentage contribution of DAB-stained cells with strong positive, positive and weak positive/negative immunoreactivity for CYP2J, CYP2C or CYP3A4 antibodies

Comparison of the absolute concentrations of EET metabolites in human tumor tissues show that they are 3- to 7-fold higher in TNBC tumors compared with the receptor positive subtypes (Fig. 2a). To construct a scale-invariant tissue classifier independent of data normalization, we compared the individual ratios of EET and corresponding DHET metabolites between normal and tumor samples per patient. The four groups of EET/DHET metabolite ratios were significantly higher in TNBC compared with the other breast cancer subtypes (*P* < 0.05) (Fig. 2b), suggesting that the EET/DHET ratio classifiers may be a promising strategy for pinpointing TNBC tumors. From these results, we hypothesized that the epoxy- derivatives of AA and expression levels of related metabolic enzymes may contribute to the TNBC phenotype.

To establish mRNA expression or tissue immunoreactivity as a proxy to metabolomics measurements, we measured the gene or protein expression levels of corresponding CYP epoxygenases in the tumor tissues. We performed qRT-PCR to compare the relative gene expressions of major EET-producing CYP epoxygenases (CYP2J2 and CYP2C19) [39, 40] and EET-metabolizing epoxide hydrolase (sEH) in the corresponding frozen tissue sections of the paired tissue samples subjected to oxylipin metabolomics analyses (Additional file 2: Figure S1). We observed a 2- to 10-fold increase in the gene expression of CYP2C19 and CYP2J2 in the triple negative mammary tumor tissues compared with the paired normal tissues. In the other subtypes, the mean difference in gene expression between paired tissue specimens was only between 1.5 and 3-fold. mRNA levels of sEH did not significantly differ between tumor and normal tissues in any of the BC specimens examined.

To obtain a more clinically relevant measure of protein expression levels, we randomly selected clinical paraffin-embedded tumor samples representative of all the breast cancer subtypes and subjected them to IHC analysis against CYP2C19/9, CYP2J2, CYP3A4 and sEH antibodies. We analyzed a total of 27 TNBC and 28 hormone receptor positive tissue specimens to determine whether the immunoreactivity can be generalized across a panel of tumor tissues collected within the same period. We assigned a three-tier scoring system for cytoplasmic stained cells in all the tissues using the automated IHC profiler plugin in ImageJ [35]. This tool utilizes a pixel-by-pixel color intensity profiling algorithm which can divide the whole tissue image equally into three staining intensity zones (strong positive, positive, and weak positive/negative) based on the arbitrary color intensity relative to the unstained background and the darkest positive DAB-stained regions. Percentile distribution for each zone is calculated for each tissue image. The tumors are classified as strong positive, positive, or weak positive/negative if the percentile score for the corresponding zone is equal to or greater than 33%. Four representative IHC images for individual CYP2J2, CYP2C9/19 and sEH enzymes, with corresponding tumor subtype and tissue classification are presented in Fig. 2c. The average percentile scores per subtype are quantified in Fig. 2d. The quantified IHC data showed that the TNBC specimens may be classified as strongly positive against CYP2J2 (58%) and strong positive/positive against CYP2C9/19 (57% strong positive and 33% positive) antibodies, respectively, which are in good agreement with the high level of EETs and CYP expoxygenase/sEH gene overexpression detected in the same tumor specimen. All ER+/PR+ specimens have weak positive/negative scores (60%) for both CYP2C9/9 and CYP2J2. HER2+ tumor tissues are classified positive or weak positive/negative against CYP2J2 (33% positive and 45% weak positive/negative), and weak positive/negative against CYPC9/19 (40%). TPBC tumors are classified weak positive/negative against CYP2J2 (64%), and positive or weak positive/negative against CYPC9/19 (49% weak positive/negative and 35% positive). Interestingly, sEH scores for ER+/PR+, HER2+, and TPBC specimens are classified as positive (46%), positive (42%) and strong positive (33%), and positive (39%), respectively, while TNBC specimens are classified as 54% weak positive/negative. These data suggest that the IHC results of CYP epoxygenase and sEH may be useful as a tool for subtyping hormone positive vs. negative BC tumors. All tissue blocks, regardless of the receptor subtype, have 31–34% positive and 43–56% weak positive/negative immunoreactivity against CYP3A4 (Additional file 2: Figure S2).

To further probe the relationship between EET metabolites, and the gene and protein expression levels of CYP2C19 and CYP2J, either detected in frozen or paraffin-embedded tumor tissues, we assembled and compared the data from the tumor samples with corresponding metabolomics, gene expression and IHC scores. Four specimens for TNBC (a), 6 specimens for ER+/PR+/Her2– (b), 5 specimens for HER2-overexpressing (c), and 5 specimens for TPBC (d) were compared (Additional file 2: Figure S3). TNBC tumors indeed had higher CYP epoxygenase mRNA and protein expression levels than the hormone positive tumors, which were reflected in the parallel elevation of EET metabolite levels. These results demonstrate that gene expression or IHC analysis of both CYP epoxygenases may be used as surrogates to explore the roles of EET signaling in breast cancers, especially in TNBC.

### EET metabolite levels are significantly elevated in human-derived mesenchymal-like TNBC cells

**Fig. 3:**
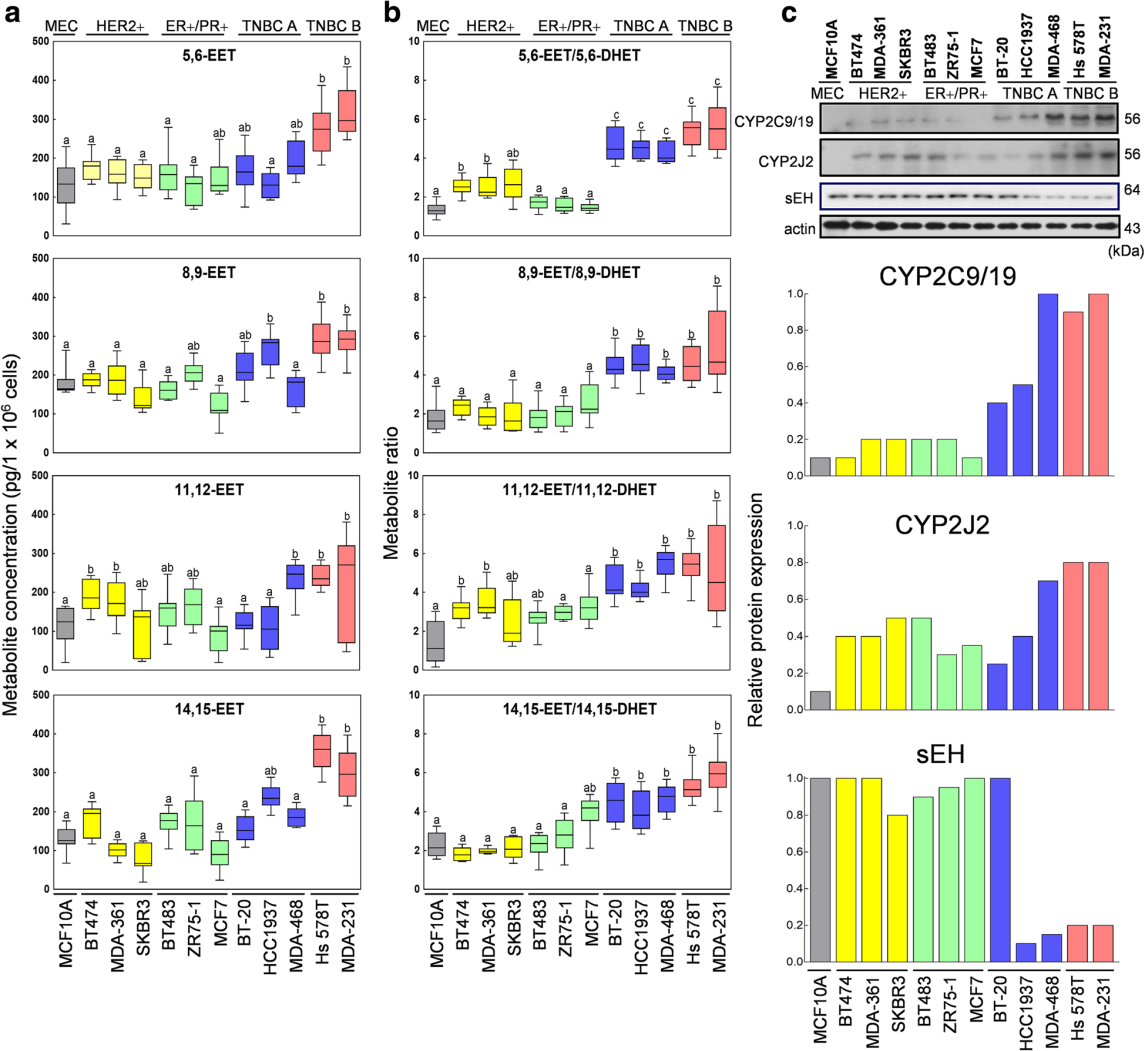
Endogeneous concentration of EETs and protein expression of related CYP epoxygenases correlates with receptor status of human-derived breast cancer cell lines. **a** Box plots show the intracellular concentration of AA-derived EETs and **b** corresponding EET/DHET metabolite ratios in human-derived TNBC (MBA-MB-231, Hs 578 T, MDA-MB-468, HCC1937 and BT-20), ER+/PR+/HER2– (MCF7, ZR75-1 and BT483), HER2-overexpressing (SKBR3, MDA-MB-361 and BT474) cell lines and normal mammary epithelial cells (MCF10A) analyzed using UPLC-MS/MS spectrometry. All analyses include 3 biological replicates and 4 technical replicates. Error bars indicate mean ± SEM. Significantly different values (*P* = 0.05, ANOVA) are denoted by letters. **c** Western blotting results show protein expression levels of CYP epoxygenases, CYP2J2 and CYP2C9/19, and sEH, in human mammary-derived cancer or normal cell lines. Representative blots of three independent experiments are shown

To further derive biological and mechanistic insights of CYPs and EETs, which were detected with significant levels from the tissue oxylipin metabolomics, immunoreactivity and gene expression analyses, we performed a series of in vitro functional assays using human-derived breast cancer cell lines representative of Her2-overexpressing (ER−/PR−/HER2+: SKBR3, MDA-MB-361 and BT474), estrogen and progesterone-expressing (ER+/PR+/HER2–; MCF7, ZR75-1 and BT483), and TNBC cells (ER−/PR−/HER2–) classified either as basal A resembling basal-like tumors (TNBC A: BT-20, HCC1937 and MDA-MB-488) or basal B displaying mesenchymal and stem-cell characteristics (TNBC B: MDA-MB-231 and Hs 578 T) [41–43]. MCF10A was utilized as a surrogate for normal mammary epithelial cells. This panel of cells has been widely used to investigate breast cancer pathobiology and is representative of the different genetic backgrounds for assessment of mechanistic or molecular comparisons parallel to clinical breast cancer cases [42]. First, we compared the intracellular levels of the four regioisomeric EET metabolites as well as their DHET products in quiescent cells (Fig. 3a). While the ratio of EETs against their corresponding downstream metabolites DHETs was significantly higher in TNBC cells regardless of sub-classification (Fig. 3b), concentrations of all EET regioisomers were significantly elevated in mesenchymal-like TNBC cells when compared with the luminal hormone receptor positive subtypes and basal-like TNBC A cells. Moreover, protein expression of CYP2C9/19 and CYP2J2 epoxygenases was significantly higher in claudin-low, mesenchymal-like TNBC cells (MDA-MB-231 and Hs 578 T) compared with all luminal (BT474, MDA-MB-361, SKBR3, MCF7, ZR75-1 and BT483) and basal-like cell lines (BT-20, HCC1937 and MDA-MB-468) (Fig. 3c). Conversely, sEH protein expressions were lowest in the TNBC cells, regardless of phenotypic sub-classification. Protein expression of CYP3A4 and CYP4A2 was very low or undetectable in all the cell lines. Despite variance in specific molecular and genetic features, e.g., mutually exclusive Ras mutation, MDA-MB-231 and Hs578T are considered highly aggressive and metastatic compared with the other cell lines examined [43]. The elevated EET and CYP expressions in these two cell lines highlight the correlation between the upregulated EET/DHET metabolic axis and the metastatic mesenchymal-like signature of basal B TNBC cell lines, regardless of the differences in genetic mutational backgrounds.

### Depletion of endogenous CYP epoxygenases attenuates the metastatic phenotype of mesenchymal-like TNBC cells

**Fig. 4:**
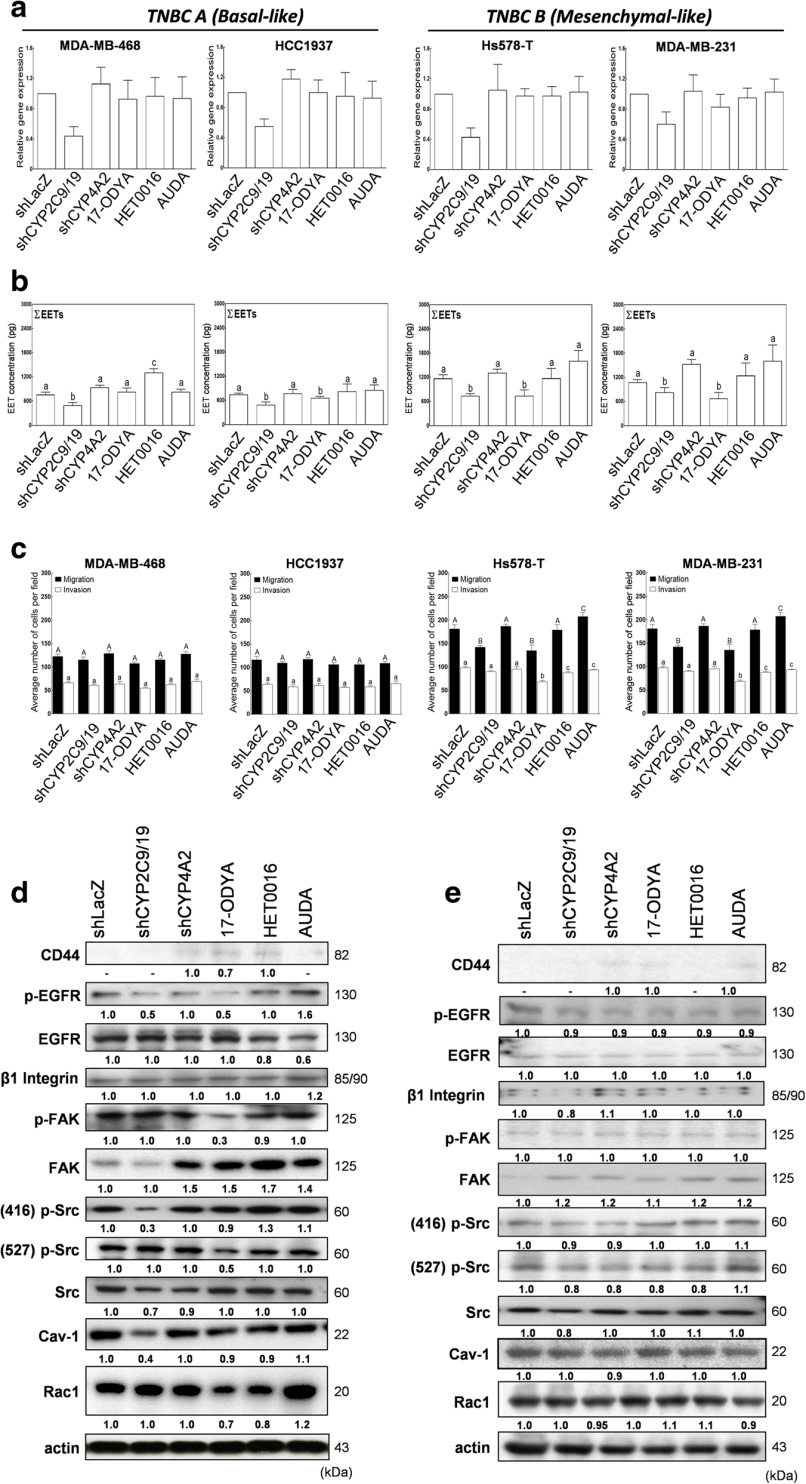
EET metabolite levels are correlated with the invasion and migration ability of basal B mesenchymal-like TNBC cells. Validation and comparison of **a** CPYP2C19 gene expression, **b** total EET concentrations and **c** invasion and migration potential among shCYP2C19 and shCYP4A2 knockdown and 17-ODYA-, HET0016- and AUDA-treated human-derived TNBC cells. Expression levels of metastasis-related proteins were significantly affected in CYP2C19-depleted or CYP-inhibitor treated **d** basal B TNBC cells (MDA-MB-231) but not in **e** basal A TNBC cells (HCC1937). All analyses include 3 biological replicates and 4 technical replicates. Error bars indicate mean ± SEM. Significantly different values (*P* = 0.05, ANOVA) are denoted by letters. All analyses include 3 biological replicates and 4 technical replicates. Error bars indicate mean ± SEM. Significantly different values (*P* = 0.05, ANOVA) are denoted by letters. Capitalized letters in **c** are for the migration data comparison and analysis. Representative blots of three independent experiments are shown

To comprehensively evaluate and correlate the roles of CYP expression and EET metabolite levels on TNBC cell metastasis, we performed shRNA-mediated depletion of the major EET-producing enzyme (CYP2C19) in both mesenchymal-like (MDA-MB-231 and Hs578T) and basal-like (MDA-MB-468 and HCC1937) TNBC cells. We chose the shRNA clone with transfection efficiency of 40–60% for shCYP2C19 for all cell lines examined (Fig. 4a). We first compared the total EET concentrations in CYP2C19 knockdown cells, or cells treated with 500 nM 17-ODYA (a CYP450 enzyme inhibitor known to lower EET levels in several cancer cell lines) [28], 500 nM HET0016 (a non-specific CYP inhibitor), and 500 nM AUDA (a specific sEH inhibitor) (Fig. 4b). These inhibitor concentrations were chosen to maximize enzymatic inhibition of CYP epoxygenase activities without compromising cell viability following 24 h treatment. shCYP4A2- (a major CYP hydroxylase) and shLacZ-transfected cells were used as controls. Total concentration of EET metabolite isomers was significantly lower in CYP2C19-depleted cells, as well as in 17-ODYA-treated cells, regardless of TNBC sub-classification (Fig. 4b). EET levels in HET0016-treated MDA-MB-468 cells were slightly elevated compared with the controls. AUDA did not induce significant changes in the total EET levels in any of the cell lines tested. Interestingly, the migration and invasion potential of basal A TNBC cells were not significantly affected by CYP2C19-depletion or treatment with any of the compound inhibitors (Fig. 4c). Moreover, depletion of CYP2C19 or partial inhibition of CYP epoxygenase activity by 17-ODYA treatment drastically reduced the metastatic potential of basal B mesenchymal-like TNBC cells. CYP4A2-depletion or treatment with HET0016 did not have an effect on either phenotype while AUDA slightly, but not significantly, increased migration and invasion in all the cell lines (*P* < 0.05). These data suggest that intrinsic CYP epoxygenase expression and endogenous EET produced by the cells might have important roles in the metastatic phenotype of claudin-low, mesenchymal TNBC cells.

Expression levels of several metastasis and stem-cell related proteins characteristically overexpressed in mesenchymal-like/basal B TNBC cells were further examined using Western blot analysis. As shown in Fig. 4d, phosphorylation of integrin regulated tyrosine kinases (EGFR, Src) and expression of a major metastatic membrane remodeling marker (Cav-1) were reduced in CYP2C19-depleted (shCYP2C19/9) MDA-MB-231 cells compared with the lacZ control. On the other hand, 17-ODYA-treated MDA-MB-231 cells had lower expression of the membrane stemness marker, CD44, two important membrane remodeling markers (Cav-1 and Rac1) and phosphorylation of integrin regulated tyrosine kinases (EGFR and FAK). Expression levels of these proteins were unaffected by CYP4A2-depletion and HET0016-treatment, while AUDA slightly increased expression of these protein markers. These results highlight that expression of CYP2C19/9 or inhibition of its activity is correlated with the expression of metastasis-related proteins in TNBC cells. Parallel immunoblotting results of basal A TNBC cell line (HCC1937) showed that expression levels of these proteins were not significantly affected in CYP2C19-depleted or CYP-inhibitor treated cells (Fig. 4e). Though it has been shown previously that 14,15-EET induced breast cancer cell EMT and drug resistance via activation of the integrin/FAK signaling axes [26], our findings pinpoint that CYP epoxygenase expression and all the 4 EET isomers are more relevant to the mesenchymal-like TNBC subtype. Targeting the expression or activity of CYP epoxygenases may be more effective to attenuate the metastasis burden for this TNBC subtype.

### EETs selectively promote the metastatic phenotype in mesenchymal-like TNBC cells

**Fig. 5:**
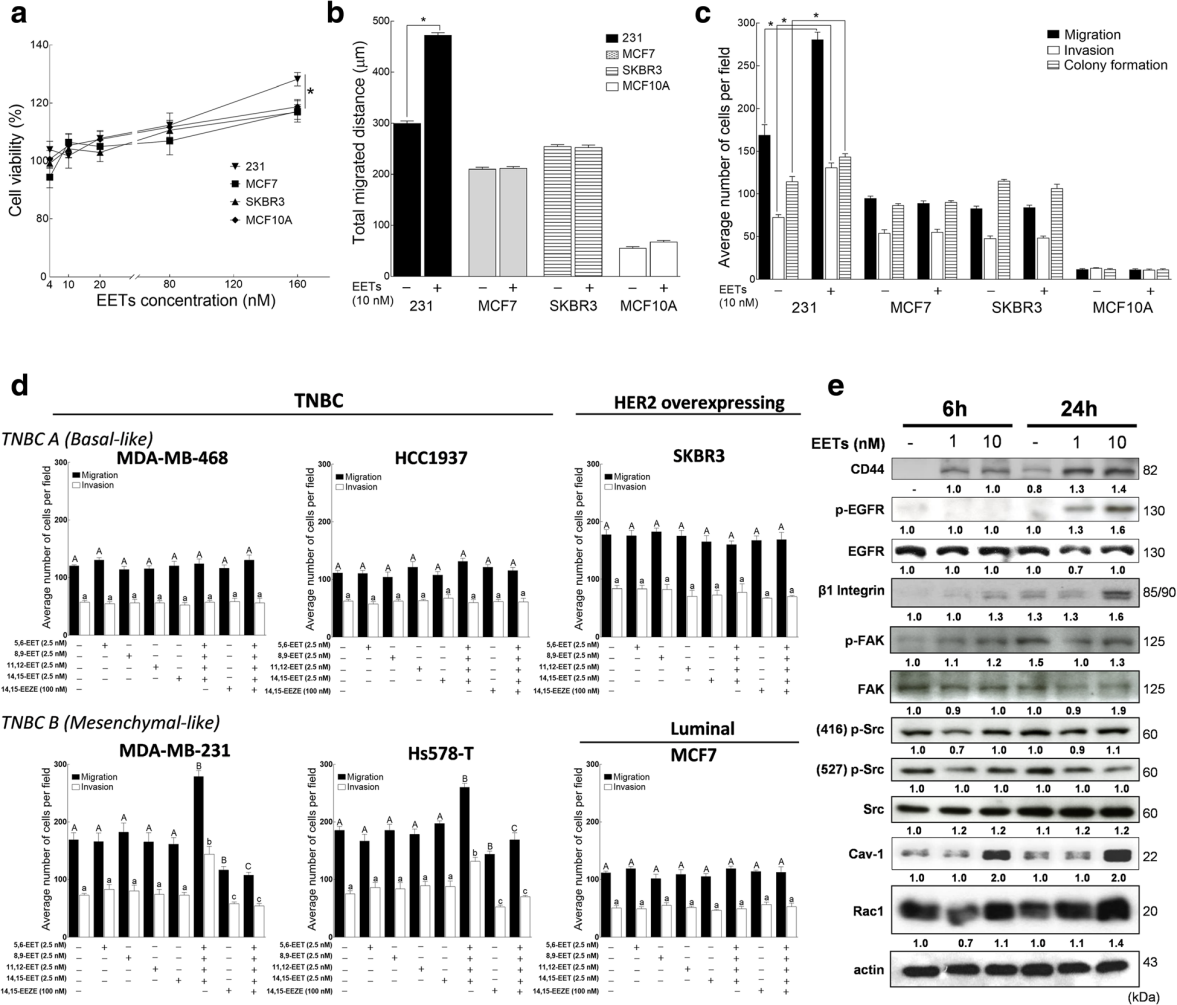
Exogenous EETs, alone or in combination, promote migration, invasion and metastasis phenotype in mesenchymal-like TNBC cells. **a** Cell viability, **b** motility, **c** migration, invasion, and colony formation following treatment with the four EET isomers in combination (cell viability: 1–40 nM each, total EET concentration of 4–160 nM; other assays: 2.5 nM each isomer, total EET concentration of 10 nM) for 24 h for representative cell lines of each breast cancer subtype were measured. **d** Increased cell migration and invasion was observed following 24 h 10 nM EET treatment, with or without 14,15-EEZE in mesenchymal TNBC cells but not in the other breast cancer cell subtypes. Error bars indicate mean ± SEM. Significantly different values (*P* = 0.05, ANOVA) are denoted by letters. All analyses include 3 biological replicates and 4 technical replicates. Error bars indicate mean ± SEM. Significantly different values (*P* = 0.05, ANOVA) are denoted by letters. Capitalized letters are for the migration data comparison and analysis. **e** Activation and expression levels of metastasis-related proteins in MDA-MB-231 cells were observed following 6 and 24 h treatment with 10 nM EETs. Representative blots of three independent experiments are shown

Next, we investigated whether EETs from exogenous sources, e.g., from stromal and infiltrating immune cells may affect the metastatic phenotype of TNBC cells. To further explore and simplify this question, e.g., eliminate the effects of immune cell-contact, cytokine-release or mechano-transduction mediated effects of co-culture experiments [44, 45], we directly supplemented cell culture media of TNBC cells with EETs. We observed that in comparison with representative hormone receptor positive cell lines examined, the viability of MDA-MB-231 cells was more significantly promoted following the addition of four EET isomers in combination (1–40 nM each isomer to make total EET concentration of 4–160 nM) (Fig. 5a). We then performed all functional assays using 10 nM of EETs, 2.5 nM of each isomer. At this concentration, exogenous EETs increased the motility of MDA-MB-231 cells as indicated by the total migrated distance measured using a time lapse live cell microscopy motility assay (Fig. 5b), migration, invasion and colony formation based on Boyden chamber and anchorage-independent clonogenic assays with *P* < 0.05 (Fig. 5c), while little effect was detected in the other three cell lines examined. Of note, individual EET at 2.5 nM did not sufficiently induce pro-metastatic behavior in MDA-MB-231 cells (Fig. 5d). The expression level of several metastasis-related proteins in the MDA-MB-231 cells with or without EET treatment were further examined using Western blotting. As shown in Fig. 5e, mesenchymal and metastatic TNBC markers CD44, Cav-1, Rac1, EGFR, Src and FAK were increased in 10 nM EETs-treated cells in a time and dose-dependent manner. A three-dimensional on top culture system was utilized to evaluate the effects of exogenous EETs on acini and metastatic phenotype formation of TNBC cells (Additional file 2: Figure S4). The globular acinar architecture was remarkably disrupted in MDA-MB-231 cells after continuous treatment with EETs (12 days). Moreover, expression of flotillin-1 and p-Src, proteins associated with membrane re-organization and metastasis transformation, were increased in EET-treated TNBC cells in comparison with MCF10A. This evidence further shows that EETs selectively promote the metastatic phenotype of TNBC cells and that EETs from exogenous sources may complement the intrinsic capacity of TNBC cells to produce EETs.

We then investigated whether these effects may be generalized for all the TNBC cell lines by comparing the invasion and migration potential of cells treated with individual EET isomers at 2.5 nM, 10 nM of EETs adding 2.5 nM of each isomer, 14,15-EEZE (a putative EET receptor antagonist) at 100 nM, and cells treated with 10 nM of EETs and 100 nM 14,5-EEZE in combination (Fig. 5d). Cells cultured in media without exogenous EETs were used as a negative control. Of note, migration and invasion potential of HER2 overexpressing and luminal cell lines were not affected by any of the treatments. The metastatic potential of MDA-MB-468 and HCC1937 were not significantly affected by any of these treatments either, while addition of 10 nM EETs significantly induced migration and invasion in MDA-MB-231 and Hs578T cells. Addition of 100 nM 14,15-EEZE abolished the effect of pro-migratory and invasion-inductive effects of 10 nM EETs in both cell lines. These results recapitulate the potential of inhibiting either the enzymatic production of EETs or its receptor(s) as a means for attenuating downstream signaling pathways contributing to the aggressive nature TNBC subtypes with mesenchymal-like signatures.

### CYP-epoxygenase expression correlates with decreased overall survival in TNBC patients

To further explore the wider clinical implications of EETs in TNBC pathology, we analyzed the correlation between mRNA expressions of EET metabolic enzymes and patient survival in silico. Kaplan–Meier analysis using data downloaded from kmplotter.com [32] showed a correlation between overexpression of major CYP epoxygenases (CYP2J2 and CYP2C19) and CYP hydroxylases (CYP2B6 and CYP4A2), CYP epoxygenase with preference for xenobiotic compounds (CYP3A4), epoxide hydrolase (EPHX3) and TNBC patient survival (Additional file 2: Figure S5). A strong association between decreased survival and overexpression of CYP2J2 (red line) (*P* = 0.0007) in TNBC patients was seen, while a weak or inverse association was observed for other receptor positive subtypes. Overexpression of CYP2C19 in TNBC patients showed a less pronounced correlation to survival rate (*P* = 0.1295). Interestingly, patients with CYP2C19-overexpressing ER+/PR+/HER2– or ER−/PR−/HER2+ tumors had better survival prospects (*P* = 0.0025 and 0.0224). An inverse relationship (*P* = 0.0123) was observed for epoxide hydrolase (EPHX3), an epoxide hydrolase isoform mainly responsible for the formation of DHETs from the substrate EETs, and survival in TNBC patients but not with the other subtypes. Expression levels of CYP2B6, CYP3A4, and CYP4A2, were not significantly associated with patient survival. These results indicate that expression level of CYP epoxygenase CY2J2 is the most relevant to TNBC patient survival.

**Fig. 6:**
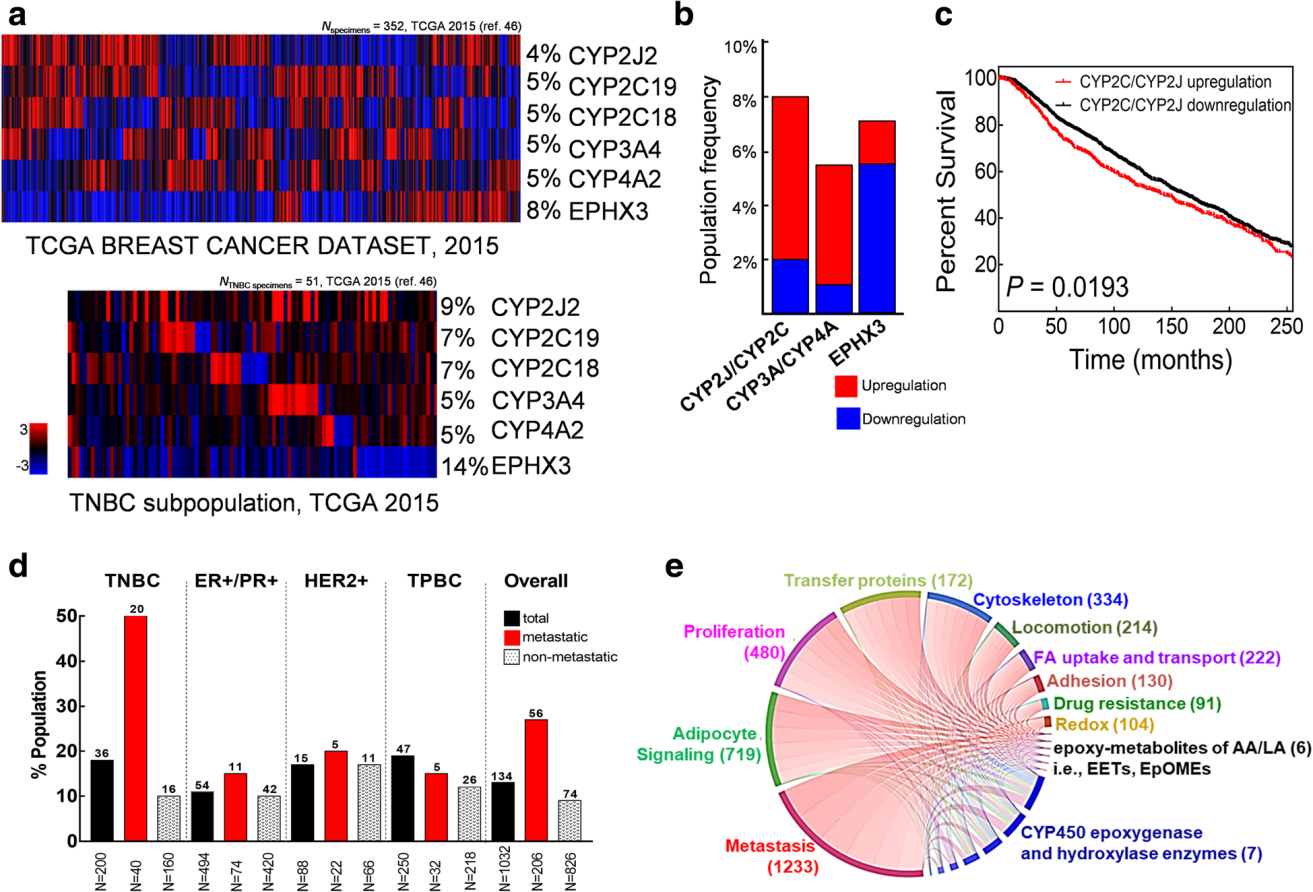
CYP epoxygenase overexpression predicts metastasis risk in TNBC patients. **a** Gene expression levels of CYP epoxygenases and hydroxylases, and soluble epoxide hydrolase related to EET biosynthesis and degradation in the overall and TNBC subset of TCGA specimen. **b** CYP epoxygenase and hydroxylase, and soluble epoxide hydrolase mRNA expression levels in the METABRIC dataset show a similar pattern to that of the TCGA dataset. **c** Overall survival plot of the TNBC subset within the METABRIC and TCGA datasets based on mRNA expression levels of CYP2C and CYP2J epoxygenase families. **d** Population distribution of patients with concurrent CYP epoxygenase upregulation and EPHX3 downregulation within the TCGA discovery set. **e** Circle plot showing direct unique network relationships of CYP epoxygenase and hydroxylase overexpression, elevated epoxy-metabolites of AA and LA and gene ontology cancer-related biological processes. The cut off value of significant relationships was set by the Benjamini-Hochberg procedure (*FDR* < 0.01)

We further explored the relationship between CYP expression levels and survival using gene expression data from an independent set of TGCA BC specimens [45]. As shown in Fig. 6a, approximately 4–5% of the overall population in this cohort (*N* = 352) had CYP epoxygenase and hydroxylase upregulation while 8% had downregulated EPHX3 expression. We then analyzed the expression profile of the six enzymes in the TNBC subpopulation (*N* = 60) within this cohort. A slight increase in patient population with CYP epoxygenase overexpression (9%) and EPHX3 downregulation (14%) was observed. Interestingly, of the TNBC subpopulation, all patients with CYP epoxygenase overexpression had concurrent EPHX3 downregulation. A similar trend was observed for the TNBC subpopulation of the METABRIC data set (*N* = 101), with 6% of the TNBC subpopulation concurrently having opposing expression levels for CYP2C19 and EPHX3 (Fig. 6b). The observed decrease in overall survival of the TNBC subpopulation with these features in both the TCGA and METABRIC cohorts (*P* = 0.0193) complemented well with the data obtained from the other independent cohorts previously analyzed (Fig. 6c).

### Network association analysis highlights convergence between EET biosynthesis and expression of metastasis-related genes in TNBC

To elucidate whether EET metabolite levels, CYP epoxygenase overexpression or sEH downregulation are involved in regulating unique pathways associated with or independent from hormone-related signaling in the different breast cancer subtypes, we constructed a discovery set comprising of mRNA microarray profiles from 3 non-overlapping BC cohorts from the TCGA database [46–48]. Pre-processed data from the METABRIC was also downloaded from cBioportal and used as an independent validation set [48, 49]. For both data sets, we only included specimens with complete receptor subgroup information based on PAM50 (Prediction Analysis of Microarray 50, Prosigna) profiles, IHC-based hormone receptor signatures, and metastasis status. The numbers of specimens per BC subtype in the discovery and validation cohorts are summarized in Additional file 1: Table S4. In Fig. 6d, we show the population breakdown of the discovery set with CYP2J2 and CYP2C19 upregulation and corresponding EPHX3 downregulation. Notably, 50% of all metastatic TNBC and 27% of the overall patient cohort had such molecular features. This observation prompted us to analyze the gene enrichment associations for the TNBC specimens in the discovery set with mRNA expression *z* score of ≥2.0 for CYP2J2 and CYP2C19. A comparison of *P* values (*P* < 0.05) derived from student *t*-test and *q* values derived from Benjamini-Hochberg procedure false discovery rate (*FDR* < 0.01) were used to identify significantly enriched (overexpressed) genes based on gene ontology (GO) term and Kyoto Encyclopedia of Genes and Genomes (KEGG) enrichment analysis. Gene sets were grouped according to oncogenic signatures, cancer gene neighborhoods or gene profile modules associated with a variety of cancer processes as curated in the Broad Institute’s Molecular Signatures Database (MSigDB). Interesting relationships between the hallmarks of breast tumor growth and invasiveness were observed, highlighting that the genes related with activation of integrin and EMT signaling, e.g., Src, AKT, and cascades related with activation of transcription factors PPAR and MYC are concominantly upregulated in TNBC tissues with CYP epoxygenase upregulation (Additional file 3). The 10 most significantly enriched biological processes and associated unique genes are: metastasis (1233), adipocyte signaling (719), proliferation (480), transfer proteins (172), cytoskeleton remodeling (334), locomotion (214), fatty acid uptake and transport (222), adhesion (130), drug resistance (91) and redox signaling (104). The circle map of the biological network relationships between the 10 cancer relevant signaling and biological processes for the TNBC subpopulation is presented in Fig. 6e. Similar analyses were performed for the other subpopulations (TPBC, HER2-overexpressing, ER+/PR+/HER2–) in the discovery set with mRNA expression *z* score of ≥2.0 for CYP2J2 and CYP2C19 (Additional file 2: Figure S6 and Additional file 4). Relationships between the gene sets in these subtypes, however, cover a wider range of biological and molecular processes, e.g., nucleic acid signaling, developmental processes and membrane trafficking, compared with the TNBC subpopulation, which were more related with metastasis and fatty acid signaling and transport. Results from this set of analyses suggest that in TNBC, CYP epoxygenase upregulation and downstream EET metabolite production and signaling may drive metastasis-related processes, while this gene-metabolite signaling axis may be involved in facilitating other distinct processes in hormone signaling-dependent breast cancers.

### Upregulation of CYP epoxygenase signaling is the strongest predictor of several cancer hallmarks in breast cancer subtypes

**Fig. 7:**
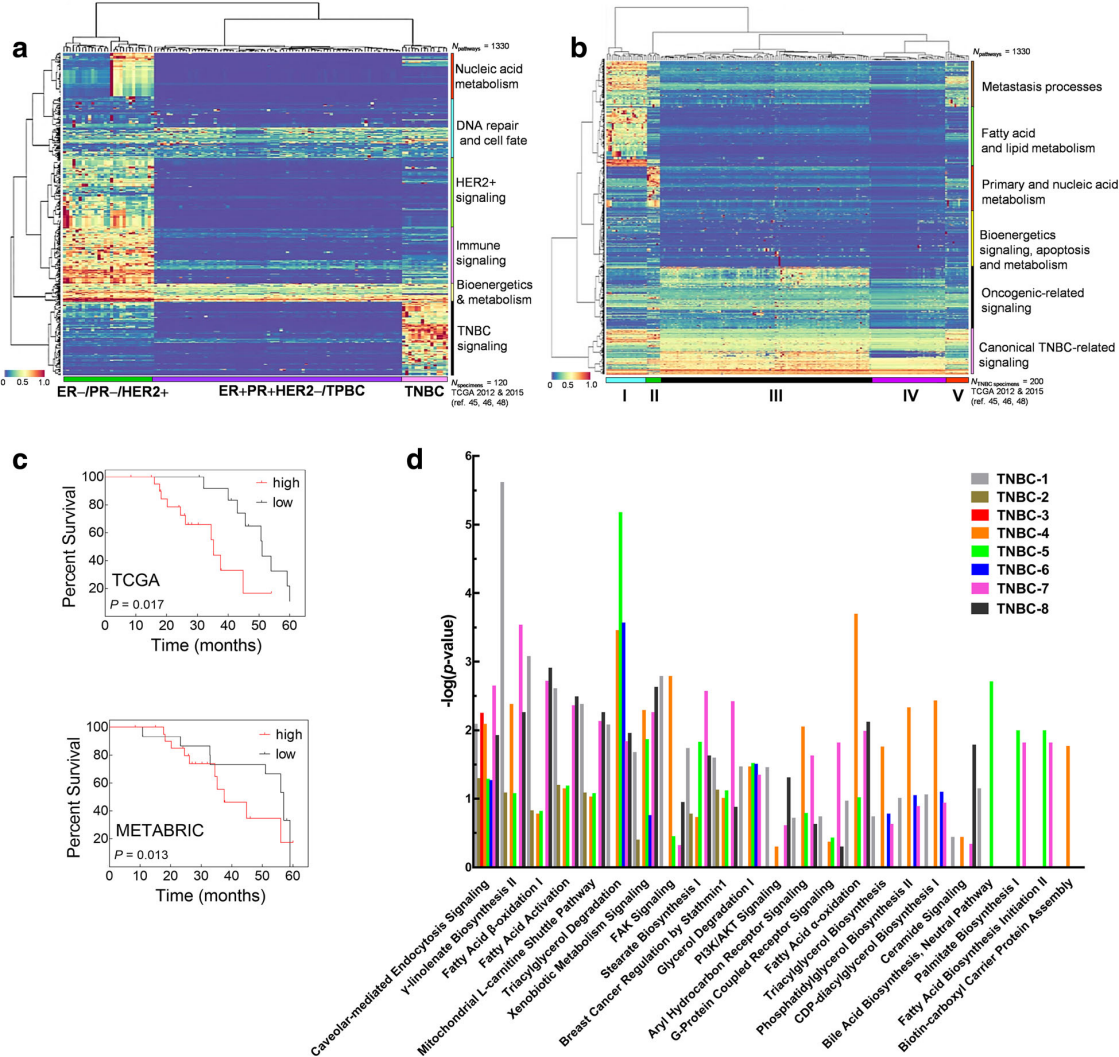
Pathway deregulation scoring (PDS) reveals targetable vulnerabilities in TNBC tissues with high CYP epoxygenase expression. **a** Hierarchical clustering of all curated pathways differentiates CYP epoxygenase overexpressing HER2+, ER+/PR+/HER2−/TPBC and TNBC tumors. **b** PDS of all TNBC samples (*N* = 200) identify a subgroup (TNBC-subtype I) with significantly higher scores for FA (arachidonic acid) metabolism and metastasis-related pathways. **c** Higher CYP epoxygenase expression is correlated with lower overall percentage survival in TNBC Subtype I patients stratified from the TCGA and METABRIC cohorts. **d** Eight TNBC samples subjected to oxylipin metabolomics and proteomics analyses consistently show upregulation of CYP epoxygenase-related gene signatures as well as metastasis-related signatures observed from the TCGA cohort. Comparatively overexpressed proteins in tumor samples were subjected to further analysis of canonical pathways using the Ingenuity Pathways Analysis (IPA) database. The canonical pathway with –log(*p*-value) ≥ 1.3 (representing *P* < 0.05) was set as a statistical significance in IPA analysis. The black dotted line indicates –log value of 1.3 as the threshold. Specimens labeled TNBC1–3 are stage IA,TNBC6–8 are stage IIB, and TNBC4–5 are stage IIIA tumor samples

To complement and strengthen the statistical associations inferred from gene and pathway level analysis, we examined the network relationships between the enriched pathways involving the genes identified as related to CYP epoxygenase upregulation. This approach may pinpoint targetable networks and result in the identification of CYP epoxygenase-modulated molecular classifiers or vulnerabilities within the breast cancer subtype. To do this, we applied Pathifier [36], an algorithm which classifies tissue specimens based on the deregulation scores (PDS) of specified signaling pathways inclusive of the genes involved in the enrichment analysis. Canonical pathways (*N* = 1330) comprising of gene set collections from the MSigDB were examined. The pathways are derived from KEGG, BioCarta, Reactome and the National Cancer Institute–Nature Pathway Interaction Database (PID). Approximately 10% of the discovery cohort had significant PDS for arachidonic acid (AA) metabolism and xenobiotic pathways, which canonically include the CYP epoxygenases (Additional file 2: Figure S7). We further hypothesized that upregulation of CYP epoxygenases may correspond to activation of several biological processes important for this subpopulation. To test this hypothesis, we constructed a PDS heat map for the subpopulation (*N* = 120) with AA and xenobiotic pathway upregulation against the whole set of canonical pathways (*N* = 1330). Figure 7a shows that the tumor specimens consistently cluster according to the breast cancer tissue classification based on receptor subtype.

We then ranked the PDS according to the *P* values and *FDR* scores. The summary of significantly upregulated pathways for each breast cancer subtype, both for the discovery (TCGA) and validation data sets (METABRIC) are listed in Additional file 1: Tables S5-S7. We uncovered commonly upregulated pathways in tumors overexpressing CYP epoxygenases regardless of hormone receptor status. These pathways are related to metabolic signaling cascades including FA beta-oxidation, mitochondrial oxidative phosphorylation and activation of glycolysis. Canonical DNA and cell fate-related events including MAPK downstream activation, cell cycle checkpoints, RNA POL II/III transcription, maintenance of chromosome integrity, as well as NOTCH, hedgehog and c-Myc transcription factor signaling were also common features among all CYP overexpressing tumors. We think that these findings are related to the known roles of lipid mediators (EETs) in the activation of transcription factor families involved in cancer cell proliferation, as well as their roles as substrates in the oxidative phosphorylation signaling cascades.

Additional file 1: Table S5 lists the 50 significantly upregulated pathways in ER−/PR−/HER2+ tumor specimens (29 tumor specimens), with the most significant pathways related to HER2 and immune-related signaling. These include pathways involved in regulation of interferon-γ (IFNG), nuclear factor-κB (NF-κB), and inflammatory interleukins/cytokines including oncogenic IL1, IL2 and IL6, STAT3, STAT5A and AKT, which are known to facilitate downstream signaling and activation of oncogenic FoxO family of transcription factors, were also identified [37, 38, 50, 51]. Most intriguingly, pathways involving the immunoglobulin superfamily of transmembrane receptors, CD28 co-stimulation, and L1CAM signaling, not previously known to be associated with HER2 signaling or CYP epoxygenases, were upregulated. As shown in Additional file 1: Table S6, twenty significantly upregulated pathways related to DNA repair and cell fate, as well as nine mutation- and metabolism-related processes were observed for the clusters comprising of ER+/PR+/HER2– and TPBC specimens (76 luminal tumor specimens). Among these, transcription factors related to chromosome maintenance, cell cycle check points, and regulation of apoptosis, including MYC, JUN, CREBBP, NCOA1 and CREB1, were highlighted. Interestingly, 62 upregulated pathways observed in the cluster comprising of TNBC specimens (15 tumor specimens) were mostly related to known TNBC-related mutations and deregulated signaling, and metastasis and membrane remodeling signaling cascades, which were not previously known to be related to CYP or AA metabolism related signaling cellular events (Additional file 1: Table S7). Genes encoding important players in cell-cell communication and junction interactions, tumor vasculation, and cell surface interactions including platelet endothelial cell adhesion molecule-1 (PECAM), vascular endothelial growth factor (VEGF), and non-receptor protein-tyrosine kinases PTK2, Src and LCK were upregulated. Upregulation of epidermal growth factor receptor (EGFR) and cascades modulated by transcription factors PPARα, PPARγ, and NCOA1 were identified to be important nodes in these processes. Our pathway-level analysis revealed that CYP-epoxygenase overexpression and activation are related to facilitating different cancer-related networks unique for each breast cancer subtype and also highlights pathways which are known to be universal drivers in breast cancer progression, e.g., MAPK, NOTCH, hedgehog and c-Myc transcription factor signaling.

We next sought to examine the critical elements in the identified pathways, which may significantly impact the design of treatment strategies for tumors with CYP upregulation. To do this, we analyzed the identified subtype-specific pathways using Webgestalt [38, 51] and performed Over Representation Analysis (ORA) and Network Topology-based Analysis (NTA). These analyses account for systems-level dependencies and interactions between genes in the pathways based on random walk network propagation which may help reveal novel and cancer-type specific co-expression networks and modules [52]. Additional file 2: Figure S8 shows the top ranking seed genes and the list of nodes related to processes upregulated in CYP epoxygenase overexpressing TNBC, HER2 and ER/PR/TPBC samples.

### CYP epoxygenase overexpression, metastatic cascade activation and low survival characterize a distinct subset of TNBC patients

We applied the same approach to determine whether the activation of metastasis-related signaling pathways maybe explored as the Achilles heel of CYP-overexpressing TNBC tumors. We constructed a PDS heat map of the canonical pathways (*N* = 1330) and all the 200 TNBC samples from the discovery set. Notably, five distinct subsets were identified, with the 62 significantly upregulated pathways characterizing the subsets within this population (Fig. 7b). Eight TNBC-related signaling cascades (Myc, Ras, MAPK and EGFR activation, HIF-1α and NOD1/2 signaling, P53 downstream cascades and ceramide metabolism) were significantly upregulated in all of the 200 TNBC tumors in the TCGA dataset. Thirty-four among the 62 pathways were metastasis related and are mainly relevant to interaction with the extracellular matrix, membrane signaling and cytoskeleton rearrangement as well as endothelial cell interactions and degradation of adherence junctions. Twenty out of the 62 pathways were related to lipid metabolism and fatty acid transformations and showcase the lipidomic phenotype of TNBC Subtype I tumors. In contrast, TNBC Subset II shows an activation of pathways related to primary metabolome and nucleic acid metabolism signaling, which seems to fuel metastatic transformation. The majority of the TNBC Subsets III-V specimens had mutations and signaling pathways known to be dysregulated in TNBC. This subtyping scheme shows that metastasis transformation of specific TNBC subsets may be driven by different endogenous metabolite cascades. We speculate that the metastatic transformation of TNBC subsets (III-V) is fueled by oncogenic signaling previously known to be associated with TNBC tumors; e.g., c-Myc and EGFR, while these processes are driven by CYP epoxygenase-mediated signaling processes in TNBC Subset I. Notably, the metastatic subpopulation (TNBC Subset I) had lower survival rates compared with low CYP epoxygenase expressing TNBC tumors (Fig. 7c). This reiterates the survival pattern seen when comparing TNBC with receptor positive subtypes, thus demonstrating the consistency and validating the relationships between TNBC, epoxygenase expression and metastasis related signal activation. These results also suggest that targeting the vulnerabilities identified using ORA and NTA (Additional file 2: Figure S7) may be more effective in CYP-overexpressing TNBC subsets that may open the door for development of personalized therapies for this subpopulation of patients.

### Convergence between EET biosynthesis and metastasis is validated using proteomics data of TNBC tumors

We then validated the conclusions drawn from pathway analysis of TCGA and METABRIC transcriptomics datasets using our own proteomics dataset. Eight paired TNBC tumor and adjacent normal tissue specimens, fragmented from the same tumor specimen used in the oxylipin metabolomics analysis were subjected to comparative proteomics using isobaric tags for relative and absolute quantitation (iTRAQ). All the peptides were filtered with a q-value threshold of 5% FDR, with the identified protein having a minimum of two unique peptides. For quantitative analysis, the relative abundance of each protein present in two biological replicates was calculated based on the iTRAQ reporter ion ratios of 115/114 and 116/114 found at the peptide level. Relative abundance of a total of approximately 3800 identified proteins is listed in Additional file 5. We annotated protein IDs to gene IDs using the Human Proteome database. We correlated the protein, gene expression levels and EET concentrations for the tissue samples and overlaid the expression values of each protein to their corresponding genes in the network. We compared the generated PDS with pathways identified from the TCGA and METABRIC datasets and observed that our proteomics data aligns well with the results derived from the large cohort-derived discovery and validation sets (Additional file 1: Table S7). We then searched for subnetworks whose expression across the patient population was highly discriminative of metastasis. Our results show that of the 8 specimens cross examined by proteomics analysis, stage IIB (*N* = 3) and IIIA tumors (*N* = 2) had higher protein expressions of CYP epoxygenase and metastasis-related genes (Fig. 7d). These results are consistent with the characteristics of the CYP overexpressing lipogenic and metastatic TNBC subtype identified via PDS analysis (Fig. 7b). Moreover, the concerted upregulation of FAK, AKT and downstream ceramide signaling are highly correlated with pathways associated with CYP epoxygenase overexpression in the discovery and validation cohorts (Additional file 1: Table S7 and Additional file 2: Figure S5). Taken together, integrative information from the tissue metabolome, proteome, and transcriptomics analyses show that EET and CYP epoxygenase expression may have broad clinical applications especially for predicting metastasis and survival, and for designing personalized therapy for TNBC patients.

## Discussion

In this study, we implemented a multi-omics, multiplatform approach using independent data sets derived from multiple cohorts and database resources, in-house collected patient samples, and a spectrum of BC cell lines to investigate specific metabolite-protein-gene pathways and signaling networks which are dysregulated or unique in different BC subtypes. The role of EETs as oncogenic metabolites specific to TNBC among the BC subtypes is highlighted by the elevated EET metabolite levels, CYP epoxygenase overexpression and sEH downregulation at the cellular level and in the TNBC tumor tissues. Of note, EET metabolite concentrations were also in good agreement with the gene and protein expression levels of CYP epoxygenases in TNBC tumor samples. Systematic cohort and bioinformatics analyses suggest that CYP epoxygenase overexpression is associated with specialized pathways dependent on BC hormone receptor status. Further, we observed that sEH protein expression is down-regulated in the TNBC tumor tissues, and thus suggest the potential role of sEH to be used as a tumor suppressor in hormone-independent BC types. We also demonstrated that this feature of TNBC tissues strongly correlates with the metastasis potential and survival outcomes as revealed by gene enrichment analyses of several publicly available transcriptomics datasets. In hormone receptor positive specimens, CYP overexpression did not have significant correlation with either the concentrations of EET metabolites in the tissues or expression of metastasis-related proteins. These results suggest that EETs may have important roles in the hormone-independent metastatic phenotype of TNBC, understanding of which may expose vulnerabilities that may be amenable to therapeutic intervention.

We used a two-tiered approach for pathway-based analysis of gene-network relationships to elucidate the biological significance of gene enrichments associated with CYP epoxygenase upregulation and to identify targetable nodes in the network. First, we used a PDS scoring strategy (Pathifier) to gauge the association between biological processes and CYP450 epoxygenase overexpression in the different breast cancer subtypes. Then we analyzed the subtype-specific pathways for systems level dependencies and pathway interactions [38, 39, 47]. In general, this approach is applicable for identification of co-expression networks and modules for populations which may be stratified according to specific molecular features. On the basis of PDS ranking, we discovered the pathways uniquely associated with HER2 overexpressing and TNBC tumor subtypes. For HER2-overexpressing tumors, these include pathways related to oncogenic signaling of interferon-γ (IFN-γ), IL1, IL2, and IL6, granulocyte-macrophage colony-stimulating factor (GM-CSF), and NF-κB. Intriguingly, pathways involving the immunoglobulin superfamily of transmembrane receptors, CD28 co-stimulation, and L1CAM signaling, not previously known to be associated with HER2 signaling or CYP epoxygenases, were upregulated. These results indicate that immune correspondence is important for HER2 positive tumors overexpressing CYP epoxygenases. It is possible that CYP epoxygenases are not expressed in HER2-overexpressing cells (as confirmed in the in vitro experiments), but are paracrine or autocrine mediators contributed by infiltrating leukocytes and other stromal cells. It would be interesting to investigate the expression of putative EET receptors and explore their roles in cancer-related signaling events in HER2 overexpressing tumors.

We have identified six TNBC-related signaling nodes, including Myc, Ras, MAPK, EGFR, HIF-1α and NOD1/2, which may be used as a gene signature for CYP epoxygenase overexpressing triple negative mammary tumors. Inhibitors or antagonists of their corresponding proteins may be considered as an approach for intervention of CYP epoxygenase overexpressing TNBC. Highly metastatic TNBC specimens may, on the other hand, be stratified according to whether they have a unique lipidomic phenotype or an upregulation of nucleic acid metabolism and signaling. Based on our network topology analyses for TNBC tissues with CYP epoxygenase upregulation, metastatic processes are highly correlated and driven by CYP epoxygenase-mediated signaling. Thus, categorizing TNBC tumors according to their EET/DHET ratio classifiers and CYP2C19 or CYP2J2 profiles, may be useful for prognostic and therapeutic assessment in TNBC. Our in vitro findings also highlight the metabolic differences between mesenchymal- and basal-like TNBC cells as well as HER2-overexpressing and luminal cell lines in terms of EET-mediated signaling and its contribution in mesenchymal-TNBC cell migration and invasion. Further probing the upstream or potential stromal regulators in TNBC may leverage the development of therapeutic agents.

## Conclusions

Taken together, our results show that the EET signaling axis regulates unique molecular cascades which are dependent on the hormone receptor status of breast tumors. Our findings highlight the importance of EETs and their surrogate epoxygenase enzymes in the metastatic behavior of TNBC cells and tumors. Integrated pathway analysis show that metastatic TNBC overexpressing CYP-epoxgenases may be most responsive to therapies targeting Myc, Ras, MAPK, EGFR, HIF-1α and/or NOD1/2. Although further studies are warranted to delineate the mechanisms underlying the identified network connections, these observations may be useful for translational applications related to prediction of survival and metastatic outcomes in TNBC patients, as well as discovery of targetable vulnerabilities and associated biomarkers for future personalized medicine applications.

## Additional files

Additional file 1: **Table S1.** Publicly available resources utilized to download and process relevant data for multi-omics network and pathway analysis. **Table S2.** Clinico-pathological characteristics of patient-derived breast tissue specimen included in the study. **Table S3.** Oxylipin metabolites identified in patient-derived mammary tumor or adjacent normal tissues. **Table S4.** Discovery and validation cohorts used for multi-omics network and pathway analysis. **Table S5.** List of pathways upregulated in histologically classified ER−/PR−/HER2+ mammary tumor samples with mRNA expression of CYP2J2 z-score ≥ 2.0. **Table S6.** List of pathways upregulated in histologically classified ER+/PR+/HER2- and TPBC tumor samples with gene expression of CYP2J2 z-score ≥ 2.0. **Table S7.** List of pathways upregulated in histologically classified TNBC tumor samples with gene expression of CYP2J2 z-score ≥ 2.0. (DOCX 98 kb)

Additional file 2: Supporting information for delineation of CYP epoxygenase-associated networks as theranostic targets for metastatic triple negative breast cancer (**Figures S1**-**S8**). (PDF 28634 kb)

Additional file 3: Gene enrichment associations for the tumor specimens in the discovery set with mRNA expression z score of ≥2.0 for CYP2J2 and CYP2C9. (XLS 7282 kb)

Additional file 4: List of nodes related to processes upregulated in CYP epoxyge nase overexpressing TNBC, ER−/PR−/HER2+ and ER+/PR+/TPBC samples. (XLS 107 kb)

Additional file 5: Quantitative proteomic data of eight paired TNBC tumors and adjacent normal tissues using iTRAQ. (XLS 3510 kb)

## Abbreviations

AA: Arachidonic acid
COX: Cyclooxygenase
CYP: Cytochrome P450
DCIS: Ductal carcinoma in situ
DHET: Dihydroxyeicosatrienoic acids
EET: Epoxyeicosatrienoic acid
EGFR: Epidermal Growth Factor Receptor
EpOME: Epoxyoctadecenoic acids
ER: Estrogen receptor
FDR: False discovery rate
GO: Gene ontology
HER2: Human epidermal growth factor receptor
HETE: Hydroxyeicosatetraenoic acids
HIF-1α: Hypoxia-inducible factor-1alpha
IHC: Immunohistopathological
iTRAQ: Isobaric tags for relative and absolute quantitation
KEGG: Kyoto Encyclopedia of Genes and Genome
LOX: Lipoxygenase
LT: Leukotriene
MAPK: Mitogen-activated protein kinase
METABRIC: Molecular Taxonomy of Breast Cancer International Consortium
MSigDB: Molecular Signatures Database
NOD 1/2: Nucleotide-binding oligomerization domain-containing protein 1
NTA: Network Topology-based Analysis
ORA: Over Representation Analysis
PAM50: Prediction Analysis of Microarray 50
PGE_2_: Prostaglandin E_2_
PID: Pathway Interaction Database
PLS-DA: PLS Discriminant Analysis
PR: Progesterone receptor
sEH: Soluble epoxide hydrolase
TCGA: The Cancer Genome Atlas
TNBC: Triple negative breast cancer
UPLC-MS/MS: Ultra-performance liquid chromatography- tandem mass spectrometry
WebGestalt: WEB-based GEne SeT AnaLysis Toolkit

## References

[1] Goldhirsch A, Winer EP, Coates AS, Gelber RD, Piccart-Gebhart M, Thurlimann B, Senn HJ (2013). Panel members: personalizing the treatment of women with early breast cancer: highlights of the St Gallen international expert consensus on the primary therapy of early breast Cancer 2013. Ann Oncol.

[2] Hu Z, Fan C, Oh DS, Marron JS, He X, Qaqish BF (2006). The molecular portraits of breast tumors are conserved across microarray platforms. BMC Genomics.

[3] Perou CM, Parker JS, Prat A, Ellis MJ, Bernard PS (2010). Clinical implementation of the intrinsic subtypes of breast cancer. Lancet Oncol.

[4] Weigelt B, Reis-Filho JS (2010). Molecular profiling currently offers no more than tumour morphology and basic immunohistochemistry. Breast Cancer Res.

[5] Paquet ER, Hallett MT (2015). Absolute assignment of breast cancer intrinsic molecular subtype. J Natl Cancer Inst.

[6] Sorlie T, Wang Y, Xiao C, Johnsen H, Naume B, Samaha RR, Borresen-Dale AL (2006). Distinct molecular mechanisms underlying clinically relevant subtypes of breast cancer: gene expression analyses across three different platforms. BMC Genomics.

[7] Rody A, Karn T, Liedtke C, Pusztai L, Ruckhaeberle E, Hanker L (2011). A clinically relevant gene signature in triple negative and basal-like breast cancer. Breast Cancer Res.

[8] Badve S, Dabbs DJ, Schnitt SJ, Baehner FL, Decker T, Eusebi V (2011). Basal-like and triple-negative breast cancers: a critical review with an emphasis on the implications for pathologists and oncologists. Mod Pathol.

[9] Beger RD (2013). A review of applications of metabolomics in cancer. Metabolites.

[10] Hsu FD, Jensen K, Cheang M, Karaca G, Hu Z, Nielsen TO (2004). Immunohistochemical and clinical characterization of the basal-like subtype of invasive breast carcinoma. Clin Cancer Res.

[11] Horiuchi D, Kusdra L, Huskey NE, Chandriani S, Lenburg ME, Gonzalez-Angulo AM (2012). MYC pathway activation in triple-negative breast cancer is synthetic lethal with CDK inhibition. J Exp Med.

[12] Stirzaker C, Zotenko E, Song JZ, Qu W, Nair SS, Locke WJ (2015). Methylome sequencing in triple-negative breast cancer reveals distinct methylation clusters with prognostic value. Nat Commun.

[13] Wiegmans AP, Al-Ejeh F, Chee N, Yap PY, Gorski JJ, Da Silva L (2014). Rad51 supports triple negative breast cancer metastasis. Oncotarget.

[14] Kim S, Lee Y, Koo JS (2015). Differential expression of lipid metabolism-related proteins in different breast cancer subtypes. PLoS One.

[15] Tang X, Lin CC, Spasojevic I, Iversen ES, Chi JT, Marks JR (2014). A joint analysis of metabolomics and genetics of breast cancer. Breast Cancer Res.

[16] Hilvo M, Denkert C, Lehtinen L, Muller B, Brockmoller S, Seppanen-Laakso T (2011). Novel theranostic opportunities offered by characterization of altered membrane lipid metabolism in breast cancer progression. Cancer Res.

[17] Beloribi-Djefaflia S, Vasseur S, Guillaumond F (2016). Lipid metabolic reprogramming in cancer cells. Oncogenesis.

[18] Wang D, Dubois RN (2010). Eicosanoids and cancer. Nat Rev Cancer.

[19] Howe LR (2007). Inflammation and breast cancer. Cyclooxygenase/prostaglandin signaling and breast cancer. Breast Cancer Res.

[20] Greene ER, Huang S, Serhan CN, Panigrahy D (2011). Regulation of inflammation in cancer by eicosanoids. Prostaglandins Other Lipid Mediat.

[21] Panigrahy D, Kaipainen A, Greene ER, Huang S (2010). Cytochrome P450-derived eicosanoids: the neglected pathway in cancer. Cancer Metastasis Rev.

[22] Karkhanis A, Hong Y, Chan ECY (2017). Inhibition and inactivation of human CYP2J2: implications in cardiac pathophysiology and opportunities in cancer therapy. Biochem Pharmacol.

[23] Panigrahy D, Edin ML, Lee CR, Huang S, Bielenberg DR, Butterfield CE (2012). Epoxyeicosanoids stimulate multiorgan metastasis and tumor dormancy escape in mice. J Clin Invest.

[24] Chen C, Wei X, Rao X, Wu J, Yang S, Chen F (2011). Cytochrome P450 2J2 is highly expressed in hematologic malignant diseases and promotes tumor cell growth. J Pharmacol Exp Ther.

[25] Jernstrom H, Bageman E, Rose C, Jonsson PE, Ingvar C (2009). CYP2C8 and CYP2C9 polymorphisms in relation to tumour characteristics and early breast cancer related events among 652 breast cancer patients. Br J Cancer.

[26] Jiang JG, Chen CL, Card JW, Yang S, Chen JX, Fu XN (2005). Cytochrome P450 2J2 promotes the neoplastic phenotype of carcinoma cells and is up-regulated in human tumors. Cancer Res.

[27] Mitra R, Guo Z, Milani M, Mesaros C, Rodriguez M, Nguyen J, Luo X (2011). CYP3A4 mediates growth of estrogen receptor-positive breast cancer cells in part by inducing nuclear translocation of phospho-Stat3 through biosynthesis of (+/−)-14,15-epoxyeicosatrienoic acid (EET). J Biol Chem.

[28] Zhang B, Cao H, Rao GN (2006). Fibroblast growth factor-2 is a downstream mediator of phosphatidylinositol 3-kinase-Akt signaling in 14,15-epoxyeicosatrienoic acid-induced angiogenesis. J Biol Chem.

[29] Wei X, Zhang D, Dou X, Niu N, Huang W, Bai J, Zhang G (2014). Elevated 14,15- epoxyeicosatrienoic acid by increasing of cytochrome P450 2C8, 2C9 and 2J2 and decreasing of soluble epoxide hydrolase associated with aggressiveness of human breast cancer. BMC Cancer.

[30] Luo J, Yao JF, Deng XF, Zheng XD, Jia M, Wang YQ (2018). 14, 15-EET induces breast cancer cell EMT and cisplatin resistance by up-regulating integrin αvβ3 and activating FAK/PI3K/AKT signaling. J Exp Clin Cancer Res.

[31] Lee CA, Neul D, Clouser-Roche A, Dalvie D, Wester MR, Jiang Y (2010). Identification of novel substrates for human cytochrome P450 2J2. Drug Metab Dispos.

[32] Gyorffy B, Lanczky A, Eklund AC, Denkert C, Budczies J, Li Q, Szallasi Z (2010). An online survival analysis tool to rapidly assess the effect of 22,277 genes on breast cancer prognosis using microarray data of 1,809 patients. Breast Cancer Res Treat.

[33] Apaya MK, Lin CY, Chiou CY, Yang CC, Ting CY, Shyur LF (2016). Simvastatin and a plant galactolipid protect animals from septic shock by regulating oxylipin mediator dynamics through the MAPK-cPLA2 signaling pathway. Mol Med.

[34] Shiau JY, Chang YQ, Nakagawa-Goto K, Lee KH, Shyur LF (2017). Phytoagent deoxyelephantopin and its derivative inhibit triple negative breast cancer cell activity through ROS-mediated exosomal activity and protein functions. Front Pharmacol.

[35] Varghese F, Bukhari AB, Malhotra R, De A (2014). IHC profiler: an open source plugin for the quantitative evaluation and automated scoring of immunohistochemistry images of human tissue samples. PLoS One.

[36] Drier Y, Sheffer M, Domany E (2013). Pathway-based personalized analysis of cancer. Proc Natl Acad Sci U S A.

[37] Wang J, Duncan D, Shi Z, Zhang B (2013). WEB-based GEne SeT AnaLysis toolkit (WebGestalt): update 2013. Nucleic Acids Res.

[38] Wang J, Vasaikar S, Shi Z, Greer M, Zhang B (2017). WebGestalt 2017: a more comprehensive, powerful, flexible and interactive gene set enrichment analysis toolkit. Nucleic Acids Res.

[39] Zeldin DC (2001). Epoxygenase pathways of arachidonic acid metabolism. J Biol Chem.

[40] Kaspera R, Totah RA (2009). Epoxyeicosatrienoic acids: formation, metabolism and potential role in tissue physiology and pathophysiology. Expert Opin Drug Metab Toxicol.

[41] Jourquin J, Duncan D, Shi Z, Zhang B (2012). GLAD4U: deriving and prioritizing gene lists from PubMed literature. BMC Genomics.

[42] Hosios AM, Hecht VC, Danai LV, Johnson MO, Rathmell JC, Steinhauser ML (2016). Amino acids rather than glucose account for the majority of cell mass in proliferating mammalian cells. Dev Cell.

[43] Kao J, Salari K, Bocanegra M, Choi YL, Girard L, Gandhi J (2009). Molecular profiling of breast cancer cell lines defines relevant tumor models and provides a resource for cancer gene discovery. PLoS One.

[44] Melzer C, von der Ohe J, Hass R (2018). Enhanced metastatic capacity of breast cancer cells after interaction and hybrid formation with mesenchymal stroma/stem cells (MSC). Cell Commun Signal.

[45] Ciriello G, Gatza ML, Beck AH, Wilkerson MD, Rhie SK, Pastore A (2015). Comprehensive molecular portraits of invasive lobular breast cancer. Cell.

[46] Pereira B, Chin SF, Rueda OM, Vollan HK, Provenzano E, Bardwell HA (2016). The somatic mutation profiles of 2,433 breast cancers refines their genomic and transcriptomic landscapes. Nat Commun.

[47] Cancer Genome Atlas Network (2012). Comprehensive molecular portraits of human breast tumours. Nature.

[48] Cerami E, Gao J, Dogrusoz U, Gross BE, Sumer SO, Aksoy BA, Jacobsen A (2012). The cBio cancer genomics portal: an open platform for exploring multidimensional cancer genomics data. Cancer Discov.

[49] Gao J, Aksoy BA, Dogrusoz U, Dresdner G, Gross B, Sumer SO (2013). Integrative analysis of complex cancer genomics and clinical profiles using the cBioPortal. Sci Signal.

[50] Shi Z, Wang J, Zhang B (2013). NetGestalt: integrating multidimensional omics data over biological networks. Nat Methods.

[51] Thummel KE (2007). Gut instincts: CYP3A4 and intestinal drug metabolism. J Clin Invest.

[52] Oh K, Lee OY, Park Y, Seo MW, Lee DS (2016). IL-1β induces IL-6 production and increases invasiveness and estrogen-independent growth in a TG2-dependent manner in human breast cancer cells. BMC Cancer.

