# Supplementary material for: Integrated omics-based pathway analyses uncover CYP epoxygenase-associated networks as theranostic targets for metastatic triple negative breast cancer
Source: J Exp Clin Cancer Res. 2019 May 9;38:187. doi: 10.1186/s13046-019-1187-y (PMC6507159; doi:10.1186/s13046-019-1187-y)
Supplement: Supplementary file 1 — Table S1. Publicly available resources utilized to download and process relevant data for multi-omics network and pathway analysis. Table S2. Clinico-pathological characteristics of patient-derived breast tissue specimen included in the study. Table S3. Oxylipin metabolites identified in patient-derived mammary tumor or adjacent normal tissues. Table S4. Discovery and validation cohorts used for multi-omics network and pathway analysis. Table S5. List of pathways upregulated in histologically classified ER−/PR−/HER2+ mammary tumor samples with mRNA expression of CYP2J2 z-score ≥ 2.0. Table S6. List of pathways upregulated in histologically classified ER+/PR+/HER2- and TPBC tumor samples with gene expression of CYP2J2 z-score ≥ 2.0. Table S7. List of pathways upregulated in histologically classified TNBC tumor samples with gene expression of CYP2J2 z-score ≥ 2.0. (DOCX 98 kb) [file 13046_2019_1187_MOESM1_ESM.docx]

**Additional file 1**

**Table S1** Publicly available resources utilized to download and process relevant data for multi-omics network and pathway analysis.

| **Website** | **Information** | **Latest access date/s** |
| --- | --- | --- |
| https://tcga-data.nci.nih.gov/tcga/ | Source for the mRNA expression data from the TCGA breast cancer cohorts (2012, and 2015) | November 2018 |
| https://kmplot.com/analysis/ | Survival analysis curves derived from curate transcriptomics data sets | January 2018 |
| https://www.cbioportal.org/ | Curated of sample information and mRNA datasets derived from TCGA and METABRIC breast cancer specimens | November 2018 |
| https://bioconductor.org/packages/release/bioc/html/pathifier.html | Source codes and tutorials for R package Pathifier | November 2018 |
| https://github.com/compgenome365/TCGA-Assembler-2 | Source codes and tutorials for the R package TCGA Assembler | November 2018 |
| https://www.webgestalt.org/ | Online tool for the identification of subtype-specific pathways and over representation analysis (ORA) and network topology-based analysis (NTA) | December 2018 |

Table S2 Clinico-pathological characteristics of patient-derived breast tissue

specimen included in the study.

| **Molecular Subtype*** | **TNBC** | **TPBC** | **ER+/PR+/Her2−** | **ER−/PR−/Her2−** |
| --- | --- | --- | --- | --- |
| **Histologic classification** | |  |  |  |
| **Ductal** | 11 (100%) | 16 (88.8%) | 16 (84.2%) | 14 (100%) |
| **Lobular** | 0 | 1 (5.6%) | 3 (15.8%) | 0 |
| **Mucinous** | 0 | 1 (5.6%) | 0 | 0 |
| **Nodal Status** |  |  |  |  |
| **N1-N2** | 4 (36.3%) | 11 (61.2%) | 14 (73.7%) | 5 (35.7%) |
| **N0** | 5 (45.4%) | 5 (27.7%) | 4 (21.0%) | 6 (42.9%) |
| **Nx** | 2 (18.1%) | 2 (11.1%) | 1 (5.3%) | 3 (21.4%) |
| **Tumor Stage** |  |  |  |  |
| **II** | 9 (81.8%) | 13 (72.2%) | 10 (52.6%) | 9 (72.7%) |
| **III** | 2 (18.2%) | 5 (27.8%) | 9 (47.4%) | 5 (27.3%) |
| **Ki67 Status** | + | + | + | + |
| **Median Age (yr)** | 52 | 53 | 51 | 56 |

* Molecular subtyping using immunohistochemical staining for ER, PR, HER2, and Ki67 according to the ASCO/CAP guidelines.

**Table S3** Oxylipin metabolites identified in patient-derived mammary tumor or adjacent normal tissues.

| **Oxylipin** | **TNBC patients** | | | | | | | | **ER+/PR+/HER2− patients** | | | | | | | | | | | | | | | **ER−/PR−/HER2+ patients** | | | | | | | | | | | | | | | | **TPBC patients** | | | | | | | | | | | | | | | | | |  |
| --- | --- | --- | --- | --- | --- | --- | --- | --- | --- | --- | --- | --- | --- | --- | --- | --- | --- | --- | --- | --- | --- | --- | --- | --- | --- | --- | --- | --- | --- | --- | --- | --- | --- | --- | --- | --- | --- | --- | --- | --- | --- | --- | --- | --- | --- | --- | --- | --- | --- | --- | --- | --- | --- | --- | --- | --- | --- | --- |
|  | **Tumor** | | | | **Normal** | | | | **Tumor** | | | | | | **Normal** | | | | | | | | | **Tumor** | | | | | | | | **Normal** | | | | | | | | **Tumor** | | | | | | | **Normal** | | | | | | | | | | |  |
| ***CYP450 epoxygenases and hydroxylases***  **AA metabolites** | | | | | | | | | | | | | | | | | | | | | | | | | | | | | | | | | | | | | | | | | | | | | | | | | | | | | | | | | | |
| 5,6-EET | | 65.9 | ± | 18.4^a^ | | 2.5 | ± | 0.7^c^ | | 22.4 | ± | 7.6^bc^ | | | | 3.2 | | | | ± | | 1.0^c^ | | | 20.3 | | ± | | 9.4^bc^ | | 3.2 | | | ± | | 1.0^c^ | | 28.3 | | | | | ± | | 9.1^b^ | | | 3.8 | | | | ± | | 0.7^c^ | | |  |  |
| 8,9-EET | | 137.3 | ± | 13.3^a^ | | 4.4 | ± | 0.4^c^ | | 94.3 | ± | 16.0^b^ | | | | 3.6 | | | | ± | | 0.8^c^ | | | 112.5 | | ± | | 10.5^ab^ | | 4.1 | | | ± | | 0.7^c^ | | 113.1 | | | | | ± | | 9.4^ab^ | | | 3.7 | | | | ± | | 0.7^c^ | | |  |  |
| 11,12-EET | | 129.5 | ± | 31.9^a^ | | 2.8 | ± | 0.6^c^ | | 60.0 | ± | 10.9^b^ | | | | 3.2 | | | | ± | | 0.7^c^ | | | 34.4 | | ± | | 7.9^bc^ | | 2.9 | | | ± | | 0.5^c^ | | 36.9 | | | | | ± | | 4.7^bc^ | | | 3.4 | | | | ± | | 0.4^c^ | | |  |  |
| 14,15-EET | | 139.5 | ± | 6.1^a^ | | 3.8 | ± | 1.2^c^ | | 121.3 | ± | 14.3^ab^ | | | | 6.3 | | | | ± | | 2.7^c^ | | | 113.9 | | ± | | 7.3^b^ | | 5.2 | | | ± | | 1.1^c^ | | 120.1 | | | | | ± | | 6.6^b^ | | | 6.3 | | | | ± | | 4.1^c^ | | |  |  |
| 19-HETE | | 17.6 | ± | 1.5^b^ | | 2.3 | ± | 1.8^d^ | | 15.5 | ± | 1.2^b^ | | | | 3.2 | | | | ± | | 1.1^cd^ | | | 48.2 | | ± | | 5.3^a^ | | 1.6 | | | ± | | 0.3^d^ | | 21.1 | | | | | ± | | 10.9^b^ | | | 3.2 | | | | ± | | 2.3^cd^ | | |  |  |
| 20-HETE | | 48.4 | ± | 4.1^b^ | | 4.4 | ± | 0.6^c^ | | 50.6 | ± | 12.4^b^ | | | | 5.7 | | | | ± | | 1.5^c^ | | | 99.8 | | ± | | 15.3^a^ | | 3.0 | | | ± | | 1.1^c^ | | 29.4 | | | | | ± | | 7.6^b^ | | | 4.6 | | | | ± | | 1.1^c^ | | |  |  |
| **LA metabolites**  9,10-EpOME | | 69.7 | ± | 7.3^a^ | | 29.9 | ± | 3.1^bc^ | | 32.9 | ± | 3.2^b^ | | | | 3.7 | | | | ± | | 2.0^c^ | | | 36.5 | | ± | | 16.9^b^ | | 21.0 | | | ± | | 9.1^bc^ | | 67.3 | | | | | ± | | 17.9^a^ | | | 8.7 | | | | ± | | 6.3^bc^ | | |  |  |
| 12,13-EpOME | | 48.9 | ± | 3.2^a^ | | 13.1 | ± | 4.3^cd^ | | 43.8 | ± | 10.7^a^ | | | | 6.9 | | | | ± | | 3.2^d^ | | | 34.2 | | ± | | 13.7^abc^ | | 14.4 | | | ± | | 7.9^bcd^ | | 37 | | | | | ± | | 12.4^ab^ | | | 14.3 | | | | ± | | 2.9^bcd^ | | |  |  |
| ***Soluble epoxide hydrolase***  **AA metabolites** | | | | | | |  |  | |  |  | | |  | | | |  | | |  | |  | | |  | |  | |  | | |  | |  | |  | | | |  | | |  | |  | | | |  | | |  | |  | | | |
| 5,6-DHET | 8.4 | | ± | 1.8^c^ | | 72.6 | ± | 6.7^b^ | | 7.0 | ± | 1.6^c^ | | | | 98.2 | | | | ± | | 5.9^ab^ | | | 5.5 | | ± | | 1.8^c^ | | 102.2 | | | ± | | 22.8^a^ | | 6.6 | | | | | ± | | 1.5^c^ | | | 107.7 | | | | ± | | 16.2^a^ | | |  |  |
| 8,9-DHET | 18.8 | | ± | 3.2^d^ | | 88.8 | ± | 7.6^ab^ | | 30.1 | ± | 1.1^cd^ | | | | 91.4 | | | | ± | | 7.6^a^ | | | 70.3 | | ± | | 0.8^b^ | | 78.7 | | | ± | | 5.4^ab^ | | 43.2 | | | | | ± | | 0.6^c^ | | | 93.1 | | | | ± | | 14.1^a^ | | |  |  |
| 11,12-DHET | 19.6 | | ± | 2.4^c^ | | 60.1 | ± | 11.8^b^ | | 17.5 | ± | 1.5^c^ | | | | 79.7 | | | | ± | | 6.2^a^ | | | 10.5 | | ± | | 0.5^c^ | | 93.5 | | | ± | | 5.1^a^ | | 10.7 | | | | | ± | | 0.9^c^ | | | 87.4 | | | | ± | | 10.6^a^ | | |  |  |
| 14,15-DHET | 14.8 | | ± | 1.3^cd^ | | 90.7 | ± | 1.0^b^ | | 3.8 | ± | 1.6^d^ | | | | 85.4 | | | | ± | | 3.4^b^ | | | 21.2 | | ± | | 0.9^c^ | | 104.2 | | | ± | | 4.7^a^ | | 24.2 | | | | | ± | | 1.4^c^ | | | 87.7 | | | | ± | | 9.9^b^ | | |  |  |
| THF diols/THETs | 8.9 | | ± | 4.5^a^ | | 11.6 | ± | 7.7^a^ | | 14.6 | ± | 6.4^a^ | | | | 54.3 | | | | ± | | 12.3^b^ | | | 15.0 | | ± | | 2.9^a^ | | 21.5 | | | ± | | 7.6^a^ | | 12.6 | | | | | ± | | 3.6^a^ | | | 22.1 | | | | ± | | 9.4^a^ | | |  |  |
| **LA metabolites** |  | |  |  | |  |  |  | |  |  |  | | | |  | | | |  | |  | | |  | |  | |  | |  | | |  | |  | |  | | | | |  | |  | | |  | | | |  | |  | | |  |  |
| 9,10-DHOME | 2.3 | | ± | 0.7^c^ | | 22.2 | ± | 1.9^b^ | | 2.7 | ± | 1.6^c^ | | | | 20.9 | | | | ± | | 0.5^b^ | | | 4.2 | | ± | | 3.7^c^ | | 27.7 | | | ± | | 6.3^ab^ | | 3.0 | | | | | ± | | 2.8^c^ | | | 33.0 | | | | ± | | 1.4^a^ | | |  |  |
| 12,13-DHOME | 12.1 | | ± | 8.3^b^ | | 19.5 | ± | 3.2^ab^ | | 11.7 | ± | 4.4^b^ | | | | 29.7 | | | | ± | | 4.0^a^ | | | 12 | | ± | | 5.3^b^ | | 20.2 | | | ± | | 4.6^ab^ | | 11.4 | | | | | ± | | 8.5^b^ | | | 19.1 | | | | ± | | 5.0^ab^ | | |  |  |
| ***Cyclooxygenases***  **AA metabolites** | | | | | | | | | | | | | | | | | | | | | | | | | | | | | | | | | | | | | | | | | | | | | | | | | | | | | | | | | | |
| PGD_2_ | 36.7 | | ± | 4.6^b^ | | 13.6 | ± | 10.9^c^ | | 32.6 | ± | 8.3^bc^ | | | | 15.4 | | | | ± | | 6.0^bc^ | | | 75.0 | | ± | | 10.8^a^ | | 17.8 | | | ± | | 9.4^bc^ | | 24.7 | | | | | ± | | 3.7^bc^ | | | 13.2 | | | | ± | | 4.5^c^ | | |  |  |
| PGE_2_ | 47.1 | | ± | 3.9^b^ | | 11.0 | ± | 9.7^d^ | | 46.7 | ± | 3.2^b^ | | | | 2.4 | | | | ± | | 0.8^d^ | | | 85.7 | | ± | | 13.6^a^ | | 13.7 | | | ± | | 8.2^cd^ | | 32.2 | | | | | ± | | 7.2^bc^ | | | 17.5 | | | | ± | | 3.2^cd^ | | |  |  |
| PGB_2_/PGJ_2_ | 7.9 | | ± | 2.2^ab^ | | 12.7 | ± | 10.4^ab^ | | 9.8 | ± | 4.9^ab^ | | | | 2.4 | | | | ± | | 1.2^ab^ | | | 28.3 | | ± | | 19.5^a^ | | 8.8 | | | ± | | 7.4^ab^ | | 9.0 | | | | | ± | | 5.8^ab^ | | | 6.1 | | | | ± | | 1.4^ab^ | | |  |  |
| 15-deoxy-PGJ_2_ | 48.3 | | ± | 8.4^ab^ | | 7.0 | ± | 2.3^d^ | | 53.9 | ± | 12.6^a^ | | | | 6.1 | | | | ± | | 4.1^d^ | | | 31.1 | | ± | | 8.9^bc^ | | 15.7 | | | ± | | 9.2^cd^ | | 24.6 | | | | | ± | | 8.2^cd^ | | | 11.0 | | | | ± | | 3.6^cd^ | | |  |  |
| PGF_2α_ | 18.3 | | ± | 6.2^abc^ | | 16.1 | ± | 1.8^abc^ | | 14.8 | ± | 5.2^bc^ | | | | 6.9 | | | | ± | | 3.7^c^ | | | 9.5 | | ± | | 6.3^bcc^ | | 20.5 | | | ± | | 1.8^ab^ | | 6.9 | | | | | ± | | 4.4^c^ | | | 27.5 | | | | ± | | 2.1^a^ | | |  |  |
| 6-keto-PGF_1α_ | 8.2 | | ± | 2.1^cd^ | | ND | | | | 7.4 | ± | 2.8^cd^ | | | | 15.2 | | | | ± | | 5.0^bc^ | | | 25.9 | | ± | | 1.2^ab^ | | 27.3 | | | ± | | 8.7^a^ | | 7.9 | | | | | ± | | 2.1^cd^ | | | 3.9 | | | | ± | | 1.3^d^ | | |  |  |
| TXB_2_ | 3.3 | | ± | 1.6^cd^ | | 17.8 | ± | 1.6^bc^ | | 21.6 | ± | 10.8^b^ | | | | 9.6 | | | | ± | | 4.3^bcd^ | | | 15.1 | | ± | | 6.4^bcd^ | | 13.6 | | | ± | | 2.0^bcd^ | | 1.0 | | | | | ± | | 0.9^d^ | | | 48.4 | | | | ± | | 8.6^a^ | | |  |  |
| ***5-LOX***  **AA metabolites** | | | | | | | | | | | | | | | | | | | | | | | | | | | | | | | | | | | | | | | | | | | | | | | | | | | | | | | | | | |
| 5-HETE | 22.4 | | ± | 5.0^c^ | | 4.0 | ± | 0.8^d^ | | 15.3 | ± | 1.3^c^ | | | | 5.8 | | | | ± | | 1.7^d^ | | | 43.4 | | ± | | 1.1^a^ | | 3.9 | | | ± | | 2.7^d^ | | 34.3 | | | | | ± | | 3.4^b^ | | | 3.9 | | | | ± | | 2.6^d^ | | |  |  |
| 5-oxoETE | 3.1 | | ± | 1.4^b^ | | 2.1 | ± | 1.2^b^ | | 7.2 | ± | 3.7^b^ | | | | 5.5 | | | | ± | | 1.8^b^ | | | 35.6 | | ± | | 14.5^a^ | | 2.0 | | | ± | | 1.1^b^ | | 5.6 | | | | | ± | | 4.9^b^ | | | 2.7 | | | | ± | | 1.7^b^ | | |  |  |
| LTA_4_ | 21.6 | | ± | 9.5^bc^ | | 34.4 | ± | 7.4^b^ | | 73.7 | ± | 16.4^a^ | | | | 5.4 | | | | ± | | 4.2^c^ | | | 37.2 | | ± | | 2.7^b^ | | 22.4 | | | ± | | 4.2^bc^ | | 85.0 | | | | | ± | | 8.9^a^ | | | 23.3 | | | | ± | | 9.8^bc^ | | |  |  |
| LTB_4_ | 43.1 | | ± | 10.5^ab^ | | 28.3 | ± | 5.0^bc^ | | 51.1 | ± | 15.8^ab^ | | | | 7.2 | | | | ± | | 4.7^c^ | | | 62.3 | | ± | | 17.2^a^ | | 28.6 | | | ± | | 2.1^bc^ | | 49.0 | | | | | ± | | 14.2^ab^ | | | 23.7 | | | | ± | | 7.^bc^ | | |  |  |
| **LA metabolites** |  | |  |  | |  |  |  | |  |  | |  | | | | |  | | |  | |  | | |  | |  | |  | | |  | |  | |  | | | |  | | |  | |  | | | |  | | |  | |  | | | |
| 9-HODE | 7.7 | | ± | 2.4^a^ | | 2.7 | ± | 0.7^a^ | | 12.0 | ± | 7.4^a^ | | | | 5.8 | | | | ± | | 4.9^a^ | | | 9.9 | | ± | | 4.5^a^ | | 11.3 | | | ± | | 8.5^a^ | | 5.8 | | | | | ± | | 3.7^a^ | | | 9.0 | | | | ± | | 6.8^a^ | | |  |  |
| 9-oxoODE | 2.1 | | ± | 1.9^a^ | | 0.8 | ± | 0.4^a^ | | 1.4 | ± | 1.1^a^ | | | | 2.0 | | | | ± | | 0.5^a^ | | | 1.8 | | ± | | 1.5^a^ | | 3.2 | | | ± | | 2.8^a^ | | 0.7 | | | | | ± | | 0.6^a^ | | | 1.3 | | | | ± | | 0.8^a^ | | |  |  |
| 9,12,13-triHOME | 23.1 | | ± | 8.9^a^ | | 9.5 | ± | 3.6^b^ | | 10.1 | ± | 6.4^ab^ | | | | 2.6 | | | | ± | | 1.2^b^ | | | 5.1 | | ± | | 3.1^b^ | | 3.9 | | | ± | | 3.2^b^ | | 5.7 | | | | | ± | | 3.8^b^ | | | 3.3 | | | | ± | | 2.1^b^ | | |  |  |
| 9,10,13-triHOME | 0.8 | | ± | 0.5^a^ | | 0.8 | ± | 0.6^a^ | | 1.5 | ± | 0.8^a^ | | | | 0.8 | | | | ± | | 0.2^a^ | | | 2.5 | | ± | | 1.7^a^ | | 2.0 | | | ± | | 0.9^a^ | | 1.2 | | | | | ± | | 0.7^a^ | | | 1.7 | | | | ± | | 1.4^a^ | | |  |  |
| ***12/15-LOX***  **AA metabolites** | | | | | | | | | | | | | | | | | | | | | | | | | | | | | | | | | | | | | | | | | | | | | | | | | | | | | | | | | | |
| 8-HETE | 4.6 | | ± | 1.5^c^ | | 7.9 | ± | 2.2^bc^ | | 13.2 | ± | 2.4^ab^ | | | | 13.2 | | | | ± | | 2.4^ab^ | | | 15.4 | | ± | | 2.5^a^ | | 6.7 | | | ± | | 2.4^bc^ | | 2.2 | | | | | ± | | 1.9^c^ | | | 12.5 | | | | ± | | 3.2^ab^ | | |  |  |
| 9-HETE | 3.2 | | ± | 0.5^a^ | | 4.9 | ± | 2.8^a^ | | 3.3 | ± | 1.4^a^ | | | | 6 | | | | ± | | 1.5^a^ | | | 5.4 | | ± | | 3.2^a^ | | 4.2 | | | ± | | 2.7^a^ | | 2.8 | | | | | ± | | 0.8^a^ | | | 4.5 | | | | ± | | 2.6^a^ | | |  |  |
| 11-HETE | 14.6 | | ± | 3.6^c^ | | 2.8 | ± | 1.2^cd^ | | 27.5 | ± | 8.6^b^ | | | | 5.4 | | | | ± | | 2.0^cd^ | | | 45.2 | | ± | | 4.7^a^ | | 2.9 | | | ± | | 1.9^cd^ | | 12.0 | | | | | ± | | 5.6^cd^ | | | 2.3 | | | | ± | | 1.4^d^ | | |  |  |
| 12-HETE | 27.3 | | ± | 5.9^c^ | | 1.6 | ± | 0.5^d^ | | 89.8 | ± | 2.4^a^ | | | | 5.6 | | | | ± | | 1.3^d^ | | | 42.2 | | ± | | 8.2^b^ | | 3.7 | | | ± | | 1.4^d^ | | 23.3 | | | | | ± | | 1.2^c^ | | | 4.8 | | | | ± | | 2.6^d^ | | |  |  |
| 15-HETE | 58.3 | | ± | 12.7^ab^ | | 4.7 | ± | 0.6^c^ | | 29.1 | ± | 16.3^bc^ | | | | 5.7 | | | | ± | | 0.3^c^ | | | 92.2 | | ± | | 10.3^a^ | | 4.1 | | | ± | | 0.8^c^ | | 52.1 | | | | | ± | | 34.6^ab^ | | | 4.8 | | | | ± | | 1.3^c^ | | |  |  |
| 15-oxoETE | 21.5 | | ± | 6.9^cd^ | | 3.3 | ± | 0.9^d^ | | 34.0 | ± | 12.6^bc^ | | | | | 5.8 | | | ± | | 1.2^d^ | | | 91.3 | | ± | | 15.7^a^ | | 4.5 | | | ± | | 0.7^d^ | | | 55.3 | | | | ± | | 17.7^b^ | | | | 4.7 | | | ± | | 1.2^d^ | |  |  |  |
| LXA_4_ | 0.4 | | ± | 0.2^c^ | | ND | | | | 1.1 | ± | 0.8^c^ | | | | | 1.9 | | | ± | | 0.8^c^ | | | 49.4 | | ± | | 2.2^a^ | | ND | | | | | | | | 1.0 | | | | ± | | 0.8^a^ | | | | 11.8 | | | ± | | 3.3^b^ | |  |  |  |
| LXB_4_ | 26.6 | | ± | 4.3^b^ | | 9.6 | ± | 7.0^bc^ | | 21.9 | ± | 12.2^bc^ | | | | | 2.9 | | | ± | | 1.7^c^ | | | 67.3 | | ± | | 12.6^a^ | | 18.3 | | | ± | | 8.2^bc^ | | | 19.4 | | | | ± | | 7.4^bc^ | | | | 16.6 | | | ± | | 7.2^bc^ | |  |  |  |
| **LA metabolites** |  | |  |  | |  |  |  | |  |  | |  | | | | | |  | |  | |  | | |  | |  | |  | | |  | |  | |  | | | | |  | |  | |  | | | | |  | |  | |  | | |  |
| 13-HODE | 41.3 | | ± | 24.6^a^ | | 8.6 | ± | 5.4^b^ | | 6.7 | ± | 1.9^b^ | | | | | 2.2 | | | ± | | 1.0^b^ | | | 12.1 | | ± | | 2.5^b^ | | 7.7 | | | ± | | 4.2^b^ | | | 4.7 | | | | ± | | 2.3^b^ | | | | 7.7 | | | ± | | 4.5^b^ | |  |  |  |
| 13-oxoODE | 26.8 | | ± | 10.9^ab^ | | 0.6 | ± | 0.3^c^ | | 23.7 | ± | 0.2^ab^ | | | | | 1.9 | | | ± | | 0.6^c^ | | | 30.6 | | ± | | 0.2^a^ | | 0.7 | | | ± | | 0.3^c^ | | | 17.7 | | | | ± | | 0.2^b^ | | | | 0.8 | | | ± | | 0.7^c^ | |  |  |  |

ND: Not detected

Oxylipin metabolite levels were quantified from paired breast tumors and adjacent normal mammary tissues collected during the same surgical procedure (*N* = 62). Data are presented as means ± SEM (*P* < 0.05, ANOVA, *post hoc* Tukey; values with different letters are significantly different).

**Table S4** Discovery and validation cohorts used for multi-omics network and pathway analysis.

| **Cohort*** | **TNBC** | **TPBC** | **ER+/PR+/Her2−** | **ER−/PR−/Her2+** |
| --- | --- | --- | --- | --- |
| TCGA dataset 1 (ref. 45) | 60 | 94 | 186 | 22 |
| TCGA 2 dataset 2 (ref. 46) | 51 | 62 | 210 | 29 |
| TCGA dataset 3 (cbioPortal; ref. 48) | 89 | 94 | 98 | 37 |
| METABRIC (ref. 49) | 101 | 126 | 247 | 94 |
| In-house patient-derived tissue samples | 11 | 18 | 19 | 14 |

*Gene expression datasets of tumor samples from 4 BC subtypes were downloaded from TCGA and cbioPortal. Total number of tissue specimen for each subtype is presented for each independent, non-overlapping population. Network and pathway deregulation analyses were performed for the discovery (TCGA) and validation (METABRIC) cohorts in comparison with the metabolome and proteome data obtained from in house clinical samples in this study.

**Table S5** List of pathways upregulated in histologically classified ER−/PR−/HER2+ mammary tumor samples with mRNA expression of CYP2J2 *z*-score ≥ 2.0.

|  | TCGA | | METABRIC | |
| --- | --- | --- | --- | --- |
| Pathway | *p*-value | FDR | *p*-value | FDR |
| (REACTOME) FGFR Ligand Binding and Activation | 1.02E-06 | 3.55E-05 | 3.13E-03 | 4.65E-02 |
| (REACTOME) Apoptotic Cleavage of Cellular Proteins | 1.53E-06 | 3.55E-05 |  |  |
| (BIOCARTA) HSP27 Pathway | 2.13E-06 | 3.55E-05 | 1.04E-02 | 4.65E-02 |
| (BIOCARTA) Inflammation Pathway | 4.24E-06 | 4.28E-05 |  |  |
| (PID) IL27 Pathway | 4.28E-06 | 4.28E-05 |  |  |
| (PID) S1P Metastasis Pathway | 5.48E-06 | 4.56E-05 | 1.47E-02 | 4.65E-02 |
| (PID) Syndecan 4 Pathway | 7.27E-06 | 4.80E-05 |  |  |
| (REACTOME) Regulation of IFNγ Signaling | 8.10E-06 | 4.80E-05 | 4.59E-02 | 4.65E-02 |
| (REACTOME) L1CAM Interactions | 8.63E-06 | 4.80E-05 | 1.46E-02 | 4.65E-02 |
| (PID) P38-MAPK Pathway | 1.13E-05 | 5.29E-05 |  |  |
| (BIOCARTA) Cdc42-Rac Pathway | 1.16E-05 | 5.29E-05 |  |  |
| (REACTOME) Signalling by NGF | 1.31E-05 | 5.39E-05 | 2.63E-02 | 4.65E-02 |
| (BIOCARTA) TFF Pathway | 1.40E-05 | 5.39E-05 |  |  |
| (PID) Il1 Pathway | 2.12E-05 | 5.71E-05 |  |  |
| (REACTOME) GABA A Receptor Activation | 2.38E-05 | 5.71E-05 | 1.40E-02 | 4.65E-02 |
| (BIOCARTA) GABA Pathway | 2.38E-05 | 5.71E-05 | 2.26E-02 | 4.65E-02 |
| (BIOCARTA) IL7 Pathway | 2.38E-05 | 5.71E-05 |  |  |
| (PID) Endothelin Pathway | 2.41E-05 | 5.71E-05 | 1.10E-02 | 4.65E-02 |
| (REACTOME) CGMP Effects | 2.42E-05 | 5.71E-05 | 1.93E-02 | 4.65E-02 |
| (BIOCARTA) Akt Pathway | 2.44E-05 | 5.71E-05 | 4.41E-02 | 4.65E-02 |
| (BIOCARTA) Csk Pathway | 2.50E-05 | 5.71E-05 | 2.23E-02 | 4.65E-02 |
| (PID) Insulin Glucose Pathway | 2.51E-05 | 5.71E-05 | 1.18E-02 | 4.65E-02 |
| (BIOCARTA) Kreb Pathway | 3.29E-05 | 6.88E-05 |  |  |
| (REACTOME) MAPK Targets Nuclear Events Mediated by MAP Kinases | 3.36E-05 | 6.88E-05 |  |  |
| (BIOCARTA) IFG1R Pathway | 3.47E-05 | 6.88E-05 | 1.65E-02 | 4.65E-02 |
| (BIOCARTA) CXCR4 Pathway | 3.66E-05 | 6.88E-05 |  |  |
| (PID) IGF1 Pathway | 3.72E-05 | 6.88E-05 | 6.48E-03 | 4.65E-02 |
| (PID) IL8 Chemotaxis | 4.25E-05 | 7.59E-05 | 4.35E-02 | 4.65E-02 |
| (REACTOME) GRB2 Events in ERBB2 Signaling | 4.46E-05 | 7.68E-05 | 2.74E-02 | 4.65E-02 |
| (BIOCARTA) GATA3 Pathway | 4.66E-05 | 7.77E-05 | 3.83E-02 | 4.65E-02 |
| (PID) ERBB2/ERBB3 Pathway | 7.03E-05 | 1.13E-04 |  |  |
| (PID) ERBB Network Pathway | 7.49E-04 | 1.17E-03 | 4.05E-02 | 4.65E-02 |
| (KEGG) Steroid Hormone Biosynthesis | 1.12E-03 | 1.69E-03 | 4.61E-02 | 4.65E-02 |
| (PID) ERB Genomic Pathway | 1.77E-03 | 2.61E-03 | 4.65E-02 | 4.65E-04 |
| (BIOCARTA) HER2 Pathway | 7.28E-03 | 1.04E-02 | 1.29E-02 | 4.65E-02 |
| (BIOCARTA) SPRY Pathway | 8.66E-03 | 1.20E-02 | 4.51E-02 | 4.65E-02 |
| (PID) NFAT Pathway | 1.02E-02 | 1.38E-02 | 3.20E-02 | 4.65E-02 |
| (PID) Lymph Angiogenesis Pathway | 1.25E-02 | 1.64E-02 | 3.65E-02 | 4.65E-02 |
| (BIOCARTA) Leptin Pathway | 1.42E-02 | 1.82E-02 | 1.00E-02 | 4.65E-02 |
| (PID) ERBB1 Downstream Pathway | 1.70E-02 | 2.12E-02 |  |  |
| (KEGG) Cell Cycle | 2.47E-02 | 3.01E-02 |  |  |
| (KEGG) Arachidonic Acid Metabolism | 3.21E-02 | 3.76E-02 |  |  |
| (KEGG) Peroxisome | 3.23E-02 | 3.76E-02 |  |  |
| (KEGG) Fatty Acid Metabolism | 3.49E-02 | 3.97E-02 | 4.52E-02 | 4.65E-02 |
| (KEGG) Drug Metabolism Cytochrome P450 | 3.63E-02 | 4.04E-02 | 3.78E-02 | 4.65E-02 |
| (KEGG) Glycerolipid Metabolism | 3.73E-02 | 4.05E-02 |  |  |
| (KEGG) DNA Replication | 4.33E-02 | 4.60E-02 | 3.21E-02 | 4.65E-02 |
| (PID) Androgen Receptor Regulation Pathway | 4.56E-02 | 4.76E-02 |  |  |
| (KEGG) ERBB Signaling Pathway | 4.67E-02 | 4.77E-02 | 3.80E-02 | 4.65E-02 |
| (KEGG) Linoleic Acid Metabolism | 4.84E-02 | 4.84E-02 | 4.51E-02 | 4.65E-02 |

**Table S6** List of pathways upregulated in histologically classified ER+/PR+/HER2- and TPBC tumor samples with gene expression of CYP2J2 *z*-score ≥ 2.0.

|  | TCGA | | METABRIC | |
| --- | --- | --- | --- | --- |
| Pathway | *p*-value | FDR | *p*-value | FDR |
| (REACTOME) Apoptotic Cleavage of Cell Adhesion Proteins | 1.82E-06 | 4.40E-05 |  |  |
| (PID) CXCR4 Pathway | 2.92E-06 | 4.40E-05 |  |  |
| (REACTOME) G1 Phase | 3.74E-06 | 4.40E-05 | 4.54E-03 | 5.38E-03 |
| (PID) Cdc42 Pathway | 4.00E-06 | 4.40E-05 | 4.82E-03 | 5.38E-03 |
| (KEGG) DNA Replication | 4.06E-06 | 4.40E-05 |  |  |
| (KEGG) Apoptosis | 5.75E-06 | 4.40E-05 | 4.12E-03 | 5.22E-03 |
| (BIOCARTA) PTDINS Pathway | 7.73E-06 | 4.40E-05 |  |  |
| (REACTOME) mRNA Capping | 8.90E-06 | 4.40E-05 | 6.30E-04 | 2.00E-03 |
| (PID) Caspase Cascade | 8.95E-06 | 4.40E-05 | 4.06E-04 | 2.00E-03 |
| (KEGG) Nucleotide Excision Repair | 9.43E-06 | 4.47E-05 |  |  |
| (REACTOME) Myogenesis | 1.03E-05 | 4.58E-05 |  |  |
| (REACTOME) DNA Repair | 1.49E-05 | 5.51E-05 |  |  |
| (REACTOME) Meiosis | 1.55E-05 | 5.51E-05 | 6.05E-04 | 2.00E-03 |
| (REACTOME) Fatty Acyl CoA ^B^iosynthesis | 1.67E-05 | 5.64E-05 | 1.68E-02 | 1.68E-02 |
| (KEGG) Drug Metabolism and Other Enzymes | 1.67E-05 | 5.64E-05 | 1.45E-04 | 1.37E-03 |
| (KEGG) Lysosome | 2.06E-05 | 6.72E-05 | 1.88E-03 | 3.58E-03 |
| (KEGG) Proteasome | 2.15E-05 | 6.72E-05 | 3.53E-03 | 5.16E-03 |
| (REACTOME) Signaling by Notch1 | 2.55E-05 | 6.95E-05 | 1.15E-03 | 2.69E-03 |
| (PID) TRAIL Pathway | 3.90E-05 | 9.35E-05 | 2.45E-03 | 4.24E-03 |
| (REACTOME) Hormone Ligand Binding Receptors | 4.29E-05 | 9.35E-05 | 3.43E-03 | 5.16E-03 |
| (REACTOME) IFN α/β Signaling | 4.41E-05 | 9.35E-05 | 1.27E-03 | 2.69E-03 |
| (REACTOME) Triglyceride Biosynthesis | 4.51E-05 | 9.35E-05 |  |  |
| (BIOCARTA) WNT Pathway | 5.60E-05 | 1.04E-04 | 9.71E-04 | 2.64E-03 |
| (KEGG) Endocytosis | 5.88E-05 | 1.04E-04 | 1.04E-04 | 1.37E-03 |
| (KEGG) Regulation of Autophagy | 5.91E-05 | 1.04E-04 |  |  |
| (KEGG) Pyruvate Metabolism | 7.52E-05 | 1.15E-04 | 4.11E-03 | 5.22E-03 |
| (REACTOME) Peroxisomal Lipid Metabolism | 1.64E-04 | 1.80E-04 | 5.57E-03 | 5.88E-03 |
| (PID) FOXO Pathway | 1.93E-04 | 2.06E-04 | 6.27E-04 | 2.00E-03 |
| (BIOCARTA) VDR Pathway | 1.93E-04 | 2.06E-04 |  |  |

**Table S7** List of pathways upregulated in histologically classified TNBC tumor samples with gene expression of CYP2J2 *z*-score ≥ 2.0.

|  | TCGA | | METABRIC | | TNBC 1-8* | |
| --- | --- | --- | --- | --- | --- | --- |
| Pathway | *p*-value | FDR | *p*-value | FDR | *p*-value | FDR |
| (REACTOME) Cell Surface Interactions at the Vascular Wall | 4.71E-07 | 2.87E-05 | 3.71E-02 | 4.46E-02 |  |  |
| (REACTOME) Degradation of the Extracellular Matrix | 5.49E-07 | 2.87E-05 |  |  |  |  |
| (BIOCARTA) Myosin Pathway | 7.91E-07 | 2.87E-05 |  |  |  |  |
| (KEGG) Glycerolipid Metabolism | 8.24E-07 | 2.87E-05 | 7.81E-03 | 1.85E-02 | 3.28E-04 | 4.77E-03 |
| (REACTOME) Eicosanoid Ligand Binding Receptors | 9.68E-07 | 2.87E-05 | 2.65E-02 | 3.71E-02 |  |  |
| (PID) RhoA Pathway | 1.02E-06 | 2.87E-05 | 2.34E-03 | 1.64E-02 |  |  |
| (REACTOME) FGFR4 Ligand Binding and Activation | 1.14E-06 | 2.87E-05 | 9.95E-03 | 1.99E-02 |  |  |
| (BIOCARTA) ECM Pathway | 1.20E-06 | 2.87E-05 |  |  | 5.40E-04 | 5.24E-03 |
| (PID) MYC Pathway | 1.45E-06 | 4.83E-05 |  |  | 1.67E-01 | 1.82E-01 |
| (PID) Thrombin Pathway | 1.78E-06 | 3.13E-05 | 3.83E-02 | 4.46E-02 |  |  |
| (PID) FAS Pathway | 1.81E-06 | 3.13E-05 | 7.14E-03 | 1.85E-02 | 1.63E-01 | 1.82E-01 |
| (REACTOME) SHS-Related Events | 1.96E-06 | 3.13E-05 |  |  |  |  |
| (REACTOME) Signaling by BMP | 2.29E-06 | 3.16E-05 | 5.20E-03 | 1.85E-02 |  |  |
| (PID) N-cadherin Activation Pathway | 2.35E-06 | 3.16E-05 | 4.84E-02 | 4.85E-02 |  |  |
| (PID) HIF-1α Pathway | 3.01E-06 | 3.35E-05 |  |  | 6.29E-06 | 1.01E-03 |
| (REACTOME) Activation of Rac | 3.58E-06 | 3.50E-05 |  |  | 1.73E-01 | 1.82E-01 |
| (PID) E-cadherin Degradation Pathway | 3.69E-06 | 3.50E-05 | 3.00E-04 | 1.26E-02 | 1.64E-01 | 1.82E-01 |
| (KEGG) Regulation of Actin Cytoskeleton | 3.84E-06 | 3.51E-05 | 2.58E-02 | 3.71E-02 | 3.81E-04 | 4.90E-03 |
| (REACTOME) Phospholipid Metabolism | 5.04E-06 | 4.17E-05 |  |  |  |  |
| (REACTOME) Elevation of Cytosolic Ca^2+^ Levels | 5.05E-06 | 4.17E-05 | 1.97E-03 | 1.64E-02 | 1.58E-02 | 3.97E-02 |
| (KEGG) Oxidative Phosphorylation | 5.23E-06 | 4.19E-05 |  |  | 9.67E-04 | 7.45E-03 |
| (REACTOME) Glycosphingolipid Metabolism | 5.63E-06 | 4.24E-05 | 3.75E-02 | 4.46E-02 |  |  |
| (REACTOME) NOD1/2 Signaling Pathway | 6.43E-06 | 4.57E-05 |  |  |  |  |
| (REACTOME) Fatty Acid Triacylglycerol and Ketone Body Metabolism | 6.69E-06 | 4.63E-05 | 4.16E-02 | 4.49E-02 |  |  |
| (REACTOME) Cell Junction Organization | 7.10E-06 | 4.73E-05 | 1.35E-02 | 2.54E-02 |  |  |
| (BIOCARTA) PTDINS Pathway | 7.73E-06 | 4.83E-05 | 8.38E-03 | 1.85E-02 | 1.11E-03 | 7.70E-03 |
| (PID) PDGFRα Pathway | 7.84E-06 | 4.83E-05 |  |  | 1.38E-01 | 1.82E-01 |
| (KEGG) Inositol Phosphate Metabolism | 8.09E-06 | 4.83E-05 |  |  | 1.25E-02 | 3.52E-02 |
| (REACTOME) Ras Activation Upon Ca^2+^ Infux through NMDA Receptor | 8.18E-06 | 4.83E-05 |  |  |  |  |
| (REACTOME) Synthesis of Very Long Chain Fatty Acyl CoAs | 8.85E-06 | 4.73E-05 |  |  |  |  |
| (KEGG) P53 Signaling Pathway | 9.02E-06 | 5.13E-05 |  |  | 4.38E-02 | 8.15E-02 |
| (REACTOME) PI3K Cascade | 9.81E-06 | 5.34E-05 |  |  |  |  |
| (REACTOME) Arachidonic Acid Metabolism | 1.01E-05 | 5.39E-05 | 6.87E-03 | 1.85E-02 |  |  |
| (PID) Ceramide Pathway | 1.10E-05 | 5.63E-05 | 1.91E-03 | 1.64E-02 | 1.02E-02 | 3.25E-02 |
| (REACTOME) DAG and IL3 Signaling | 1.30E-05 | 6.52E-05 | 6.30E-03 | 1.85E-02 |  |  |
| (KEGG) Metabolism of Xenobiotics by Cytochrome P450 | 1.33E-05 | 6.56E-05 | 1.39E-02 | 2.54E-02 | 1.20E-02 | 3.42E-02 |
| (BIOCARTA) MAPK Pathway | 1.40E-05 | 6.77E-05 | 1.01E-03 | 1.64E-02 | 1.58E-01 | 1.82E-01 |
| (REACTOME) Integrin Cell Surface Interactions | 1.50E-05 | 6.98E-05 | 1.98E-02 | 3.41E-02 | 8.60E-03 | 2.99E-02 |
| (PID) β-Catenin Degradation Pathway | 1.89E-05 | 8.17E-05 |  |  | 7.66E-03 | 2.83E-02 |
| (REACTOME) Integrin α/β Signaling | 1.91E-05 | 8.17E-05 |  |  | 1.02E-03 | 7.45E-03 |
| (REACTOME) GPCR Downstream Signaling | 1.91E-05 | 8.17E-05 | 3.33E-02 | 4.23E-02 |  |  |
| (REACTOME) Biological Oxidations | 1.99E-05 | 8.36E-05 | 2.64E-02 | 3.71E-02 |  |  |
| (REACTOME) Integration of Energy Metabolism | 2.08E-05 | 8.58E-05 | 2.03E-02 | 3.41E-02 |  |  |
| (KEGG) Glycerophospholipid Metabolism | 2.14E-05 | 8.71E-05 |  |  |  |  |
| (REACTOME) Pyruvate Metabolism and Citric Acid TCA Cycle | 2.23E-05 | 8.90E-05 | 1.35E-03 | 1.64E-02 |  |  |
| (^B^IOCARTA) PYK2 Pathway | 2.36E-05 | 9.08E-05 | 2.93E-02 | 3.97E-02 |  |  |
| (KEGG) Adipocytokine Signaling Pathway | 2.38E-05 | 9.08E-05 |  |  | 6.58E-06 | 5.54E-05 |
| (REACTOME) Membrane Trafficking | 2.43E-05 | 9.08E-05 | 4.17E-02 | 4.49E-02 |  |  |
| (BIOCARTA) PPARα Pathway | 2.45E-05 | 9.08E-05 | 4.85E-02 | 4.85E-02 | 1.58E-07 | 1.27E-05 |
| (REACTOME) Signaling by Constitutively Active EGFR | 2.80E-05 | 5.39E-05 | 4.53E-02 | 4.75E-02 |  |  |
| (KEGG) Gap Junction | 3.23E-05 | 1.12E-04 | 7.59E-03 | 1.85E-02 | 4.92E-05 | 1.95E-04 |
| (KEGG) VEGF Signaling Pathway | 3.24E-05 | 1.12E-04 | 8.32E-03 | 1.85E-02 | 1.17E-03 | 1.82E-03 |
| (KEGG) Linoleic Acid Metabolism | 3.55E-05 | 1.13E-04 | 5.13E-03 | 1.85E-02 |  |  |
| (REACTOME) Sphingolipid Metabolism | 3.61E-05 | 1.13E-04 | 7.92E-03 | 1.85E-02 |  |  |
| (REACTOME) PI Metabolism | 3.63E-05 | 1.13E-04 | 5.96E-03 | 1.85E-02 |  |  |
| (KEGG) Fatty Acid Metabolism | 3.72E-05 | 1.13E-04 | 5.23E-03 | 1.85E-02 | 4.35E-06 | 4.97E-05 |
| (PID) Ras Pathway | 3.75E-05 | 1.13E-04 | 2.30E-02 | 3.57E-02 |  |  |
| (REACTOME) Adherens Junctions Interactions | 4.53E-05 | 1.33E-04 | 7.01E-03 | 1.85E-02 |  |  |
| (PID) FAK Pathway | 5.61E-05 | 1.59E-04 | 9.18E-03 | 1.93E-02 |  |  |
| (REACTOME) Acyl Chain Remodeling of PS | 5.61E-05 | 1.59E-04 | 4.16E-02 | 4.49E-02 |  |  |
| (REACTOME) PECAM1 Interactions | 5.74E-05 | 1.61E-04 |  |  |  |  |
| (REACTOME) Synthesis of PC | 6.91E-03 | 1.92E-02 | 2.17E-02 | 3.50E-02 |  |  |

*Eight paired TNBC tumors and adjacent normal tissues were subjected to comparative quantitative proteomics using isobaric tags for relative and absolute quantitation (iTRAQ). Protein and gene IDs were mapped using matching annotations from the Human Proteome database. PDS scores for the corresponding gene and pathway networks were generated and compared with the PDS from the TCGA and METABRIC discovery and validation data sets.
