# Supplementary material for: Integrated omics-based pathway analyses uncover CYP epoxygenase-associated networks as theranostic targets for metastatic triple negative breast cancer
Source: J Exp Clin Cancer Res. 2019 May 9;38:187. doi: 10.1186/s13046-019-1187-y (PMC6507159; doi:10.1186/s13046-019-1187-y)
Supplement: Supplementary file 2 — Supporting information for delineation of CYP epoxygenase-associated networks as theranostic targets for metastatic triple negative breast cancer (Figures S1-S8). (PDF 28634 kb) [file 13046_2019_1187_MOESM2_ESM.pdf]

Additional File 2

Figure S1. Relative gene expression of CYP epoxygenase (CYP2J2 and CYP2C19) and epoxide hydrolase (sEH) in paired normal mammary and tumor tissue specimen. Gene expression profile of **a** CYP2J2, **b** CYP2C19, and **c** seH in paired normal and tumor specimens with corresponding metabolomics data were measured by qRT-PCR analyses. All experiments were performed in triplicate. GADPH expression for each sample was used as control.

Figure S2. Representative strong positive, positive and weak positive/negative immunostaining and corresponding quantitative scoring analyses results of IHC analyses for tumor sections (N = 55) obtained against CYP3A4 antibodies. DAB-staining was used to visualize immunoreactive regions and cellular nuclei (blue) were counterstained with hematoxylin. Images were taken at 40x magnification. Quantitative scoring analyses of the immunostained tissues using IHC profiler shows the percentage contribution of DAB-stained cells with strong positive, positive and weak positive/negative immunoreactivity for CYP3A4 antibodies.

Figure S3. Comparison of CYP epoxygenase gene and protein expressions in tissues with corresponding oxylipin metabolome data. Oxylipin metabolomics, gene expression and immunohistochemical analyses show a direct relationships among total EET metabolite concentrations, CYP2C19 and CYP2J2 gene expression and IHC scores of **a** TNBC (N = 4), **b** ER+/PR+/HER2− (N = 6), **c** ER−/PR−/HER2+ (N = 5) and **d** TPBC (N = 5) tumor specimen.

Figure S4. Effects of CYP epoxygenase expression and EET supplementation on mesencymal-like TNBC cells. Metastatic phenotype of mesencymal-like TNBC B cells (MDA-MB-231) in 3D culture following treatment with the four EET isomers (2.5 nM each isomer, total EET concentration of 10 nM) in combination.

Figure S5. CYP epoxygenase overexpression predicts survival outcomes in breast cancer patients. Survival curves using a multigene classifier for CYP epoxygenases and hydroxylases in **a** TNBC (N = 178), **b** ER+/PR+/HER2− (N = 84) and **c** ER−/PR−/HER2+ tissues (N = 138) from a publicly available curated database of gene expressions, and overall survival information from GEO, EGA and TCGA (kmplot.com).

Figure S6. Biological processes and molecular functions enriched in CYP epoxygenase overexpressing specimens. GO enrichment analysis for **a** TNBC, **b** ER+/PR+/HER2−, **c** ER−/PR−/HER2+ and **d** TPBC specimens in the discovery set with mRNA expression z score of ≥ 2.0 for CYP2J2 and CYP2C9).

Figure S7. Pathway deregulation scoring (PDS) differentiates pathways regulated by CYP upregulation in breast cancer tumor subtypes. PDS for FA metabolism-related pathways were calculated for all samples downloaded from TCGA. Breast tissue specimens (N = 120) with high PDS scores for CYP450-related pathways (KEGG xenobiotic metabolism, KEGG arachidonic acid metabolism and REACTOME fatty acid metabolism) were selected for further analysis.

Figure S8. Key targetable molecular pathways altered in CYP epoxygenase overexpressing tumor specimens. Network of potential protein targets in **a** HER2+, **b** luminal (ER+/PR+/HER2- or TPBC), and **c** TNBC tumors are determined by Over Representation Analysis (ORA) and Network Topology-based Analysis (NTA) of gene enriched in the specimens examined.

Figure S1

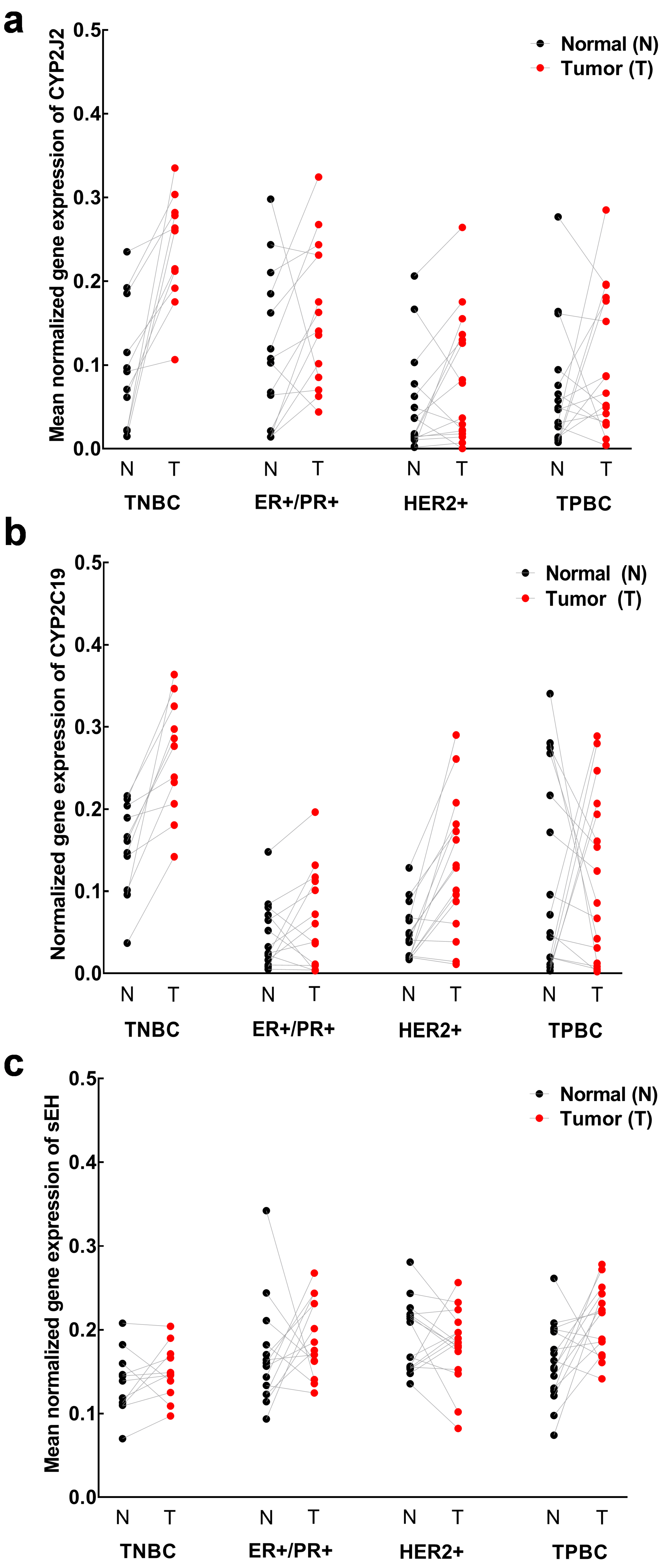
a
Normal (N)
Tumor (T)
Mean normalized gene expression of CYP2J2
0.0
0.1
0.2
0.3
0.4
0.5
N T N T N T N T
TNBC ER+/PR+ HER2+ TPBC
b
Normal (N)
Tumor (T)
Normalized gene expression of CYP2C19
0.0
0.1
0.2
0.3
0.4
0.5
N T N T N T N T
TNBC ER+/PR+ HER2+ TPBC
c
Normal (N)
Tumor (T)
Mean normalized gene expression of sEH
0.0
0.1
0.2
0.3
0.4
0.5
N T N T N T N T
TNBC ER+/PR+ HER2+ TPBC

Figure S2

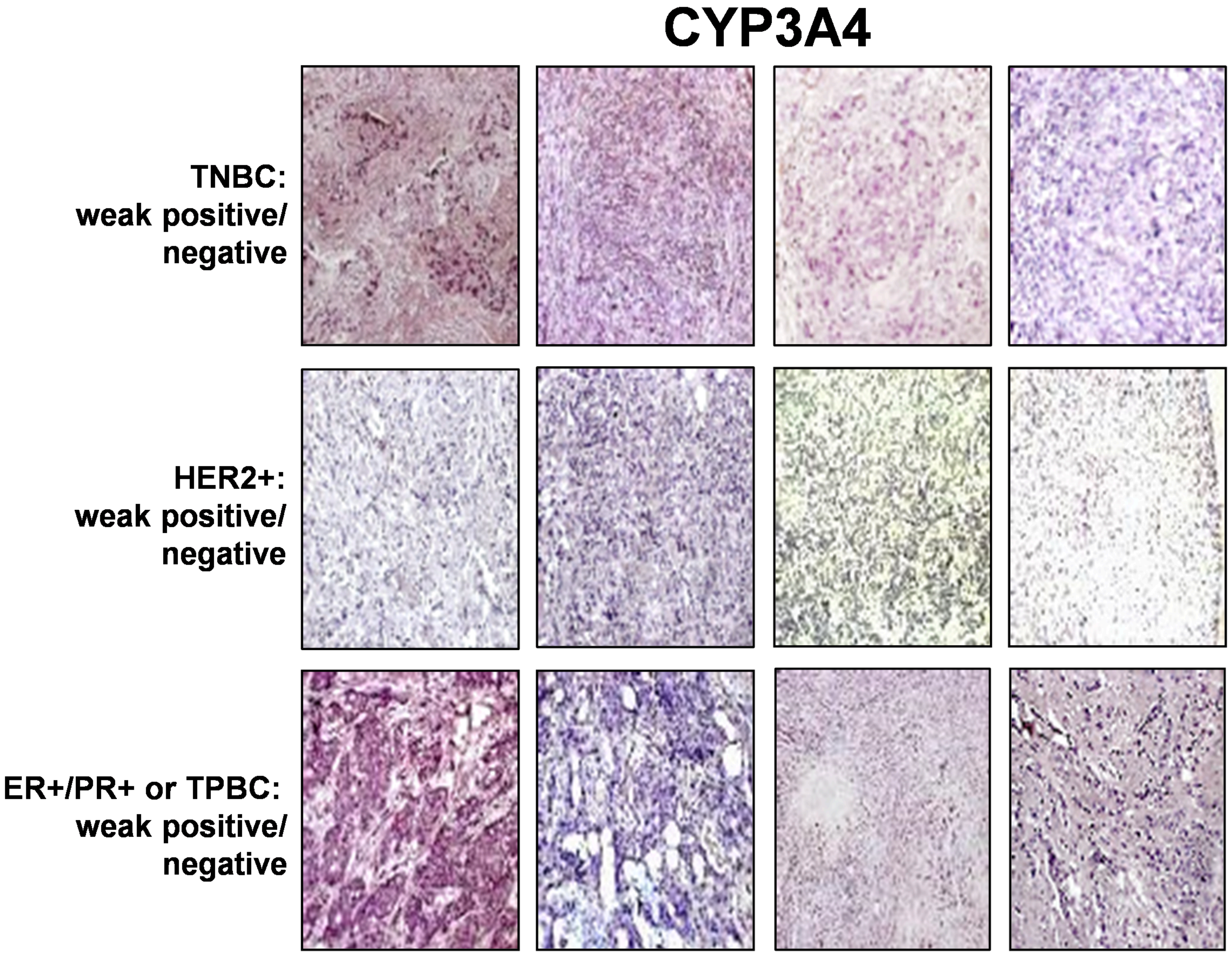
CYP3A4
TNBC:
weak positive/
negative
HER2+:
weak positive/
negative
ER+/PR+ or TPBC:
weak positive/
negative

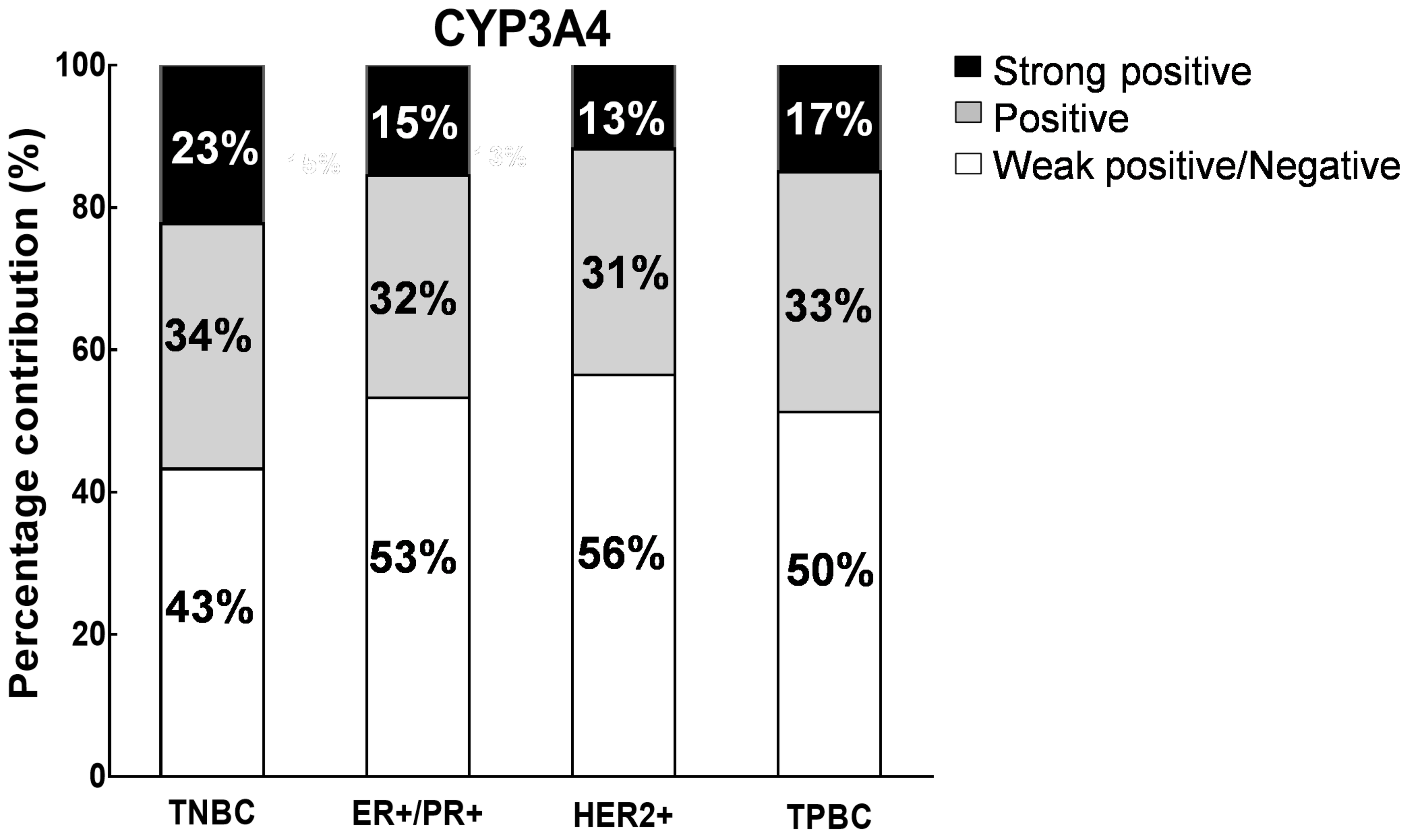
CYP3A4
Strong positive
Positive
Weak positive/Negative
Percentage contribution (%)
100
80
60
40
20
0
23%
34%
43%
15%
32%
53%
13%
31%
56%
17%
33%
50%
TNBC
ER+/PR+
HER2+
TPBC

Figure S3

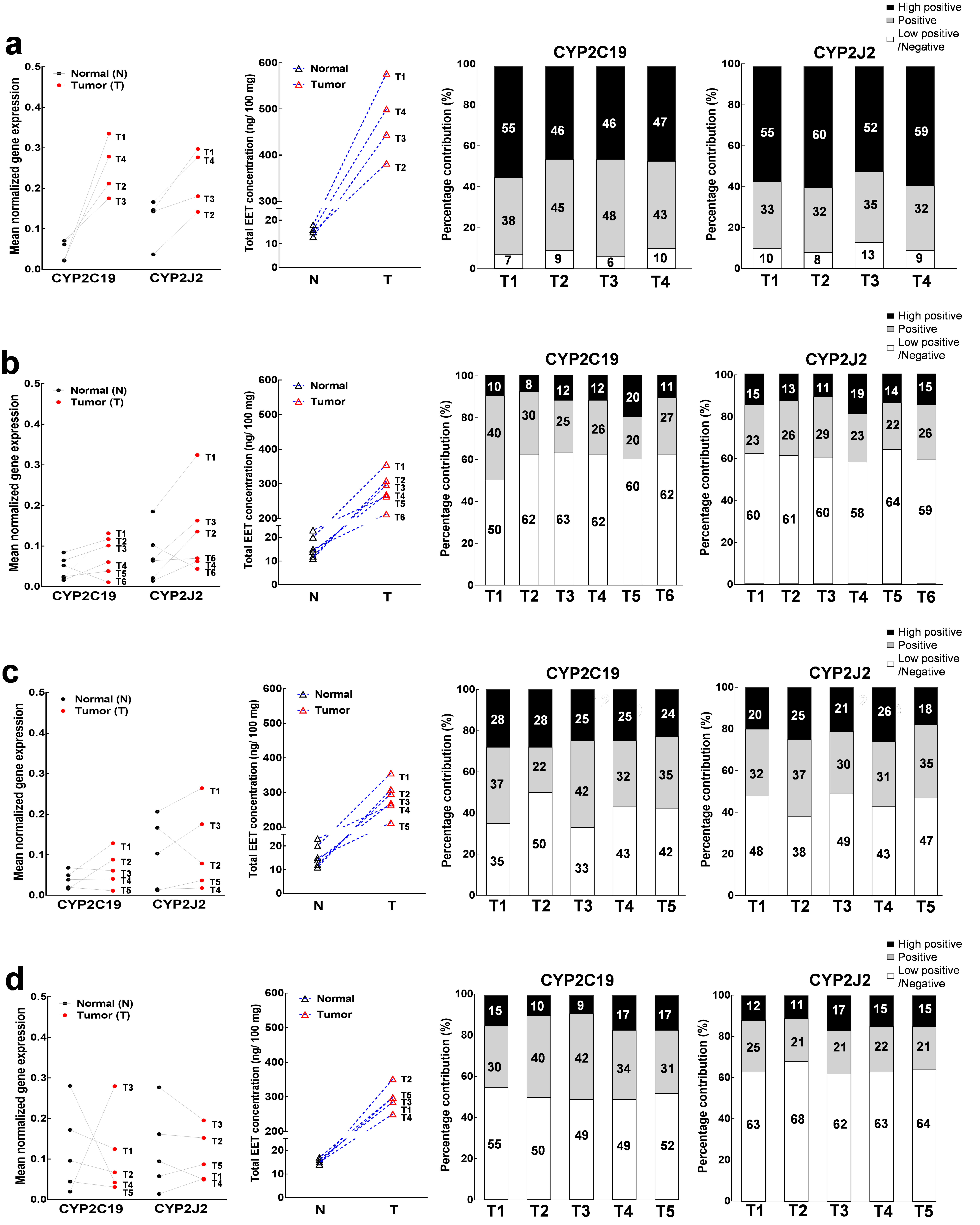

a
b
c
d
Normal (N)
Tumor (T)
Mean normalized gene expression
CYP2C19
CYP2J2
Normal
Tumor
Total EET concentration (ng/ 100 mg)
N
T
Percentage contribution (%)
High positive
Positive
Low positive /Negative
T1
T2
T3
T4
T5
T6

Figure S4

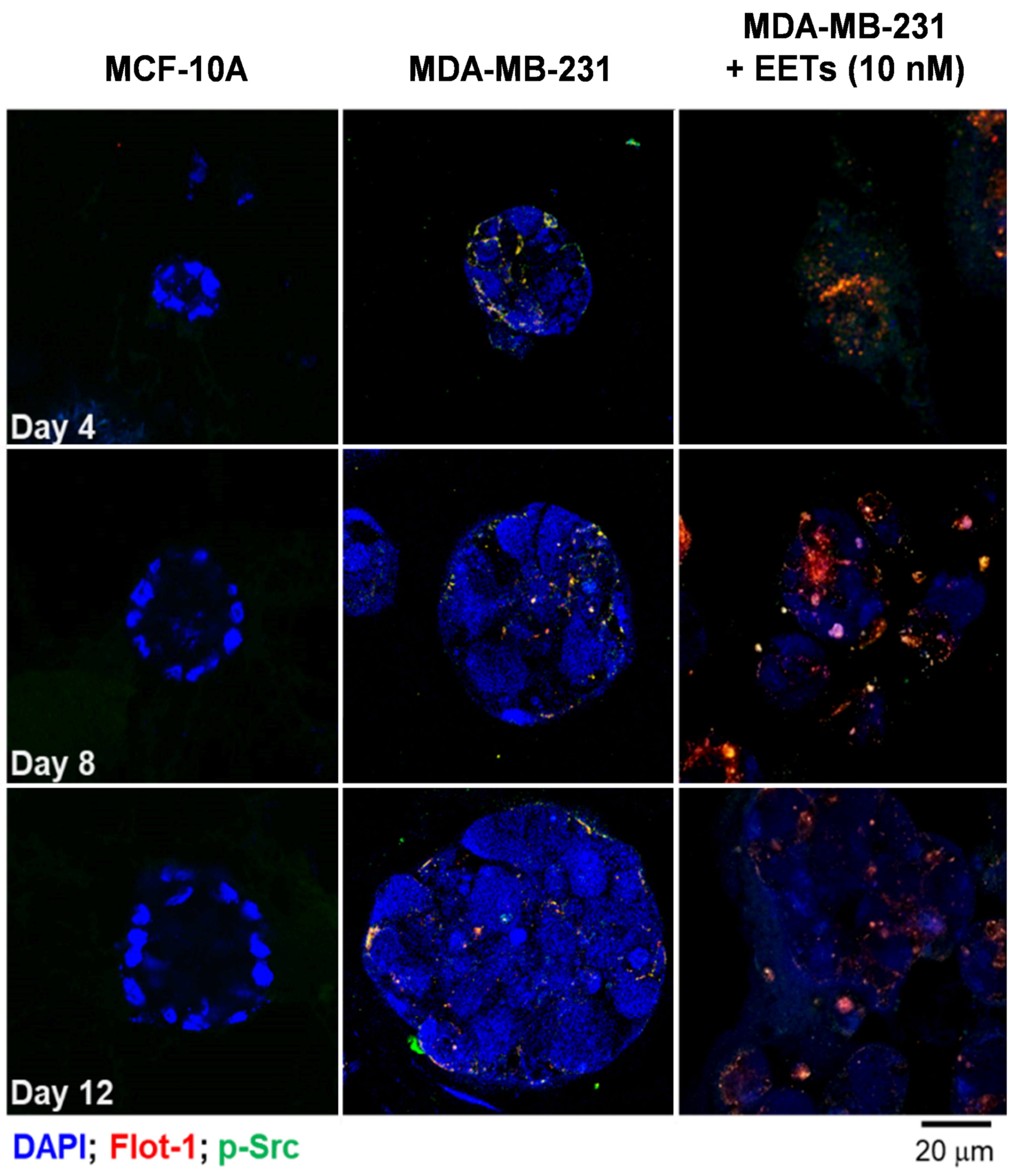

MCF-10A
MDA-MB-231
MDA-MB-231
+ EETs (10 nM)
Day 4
Day 8
Day 12
DAPI; Flot-1; p-Src
20 μm

Figure S5

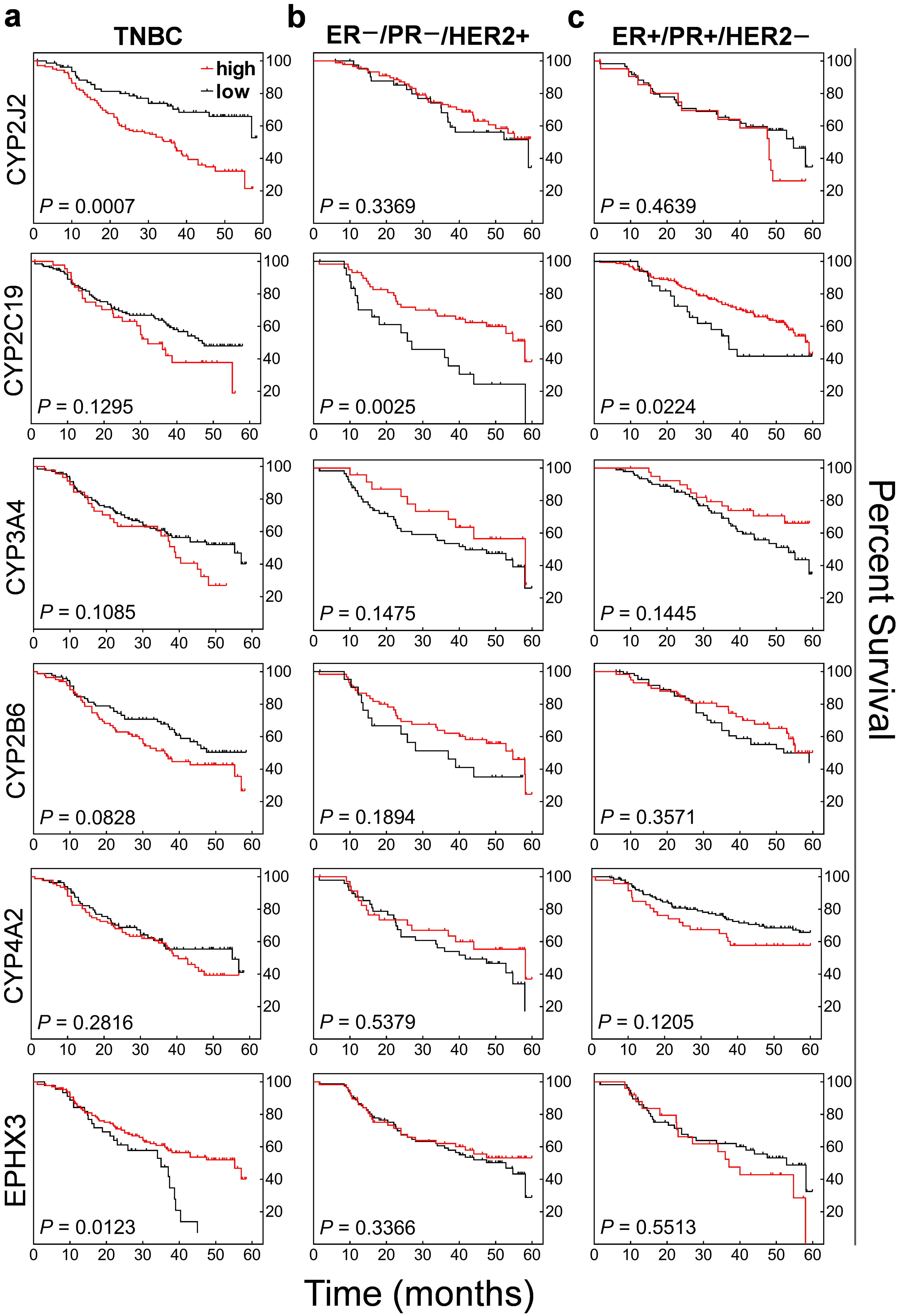

a
b
c
TNBC
ER−/PR−/HER2+
ER+/PR+/HER2−
high
low
CYP2J2
CYP2C19
CYP3A4
CYP2B6
CYP4A2
EPHX3
P = 0.0007
P = 0.3369
P = 0.4639
P = 0.1295
P = 0.0025
P = 0.0224
P = 0.1085
P = 0.1475
P = 0.1445
P = 0.0828
P = 0.1894
P = 0.3571
P = 0.2816
P = 0.5379
P = 0.1205
P = 0.0123
P = 0.3366
P = 0.5513
Percent Survival
Time (months)

Figure S6

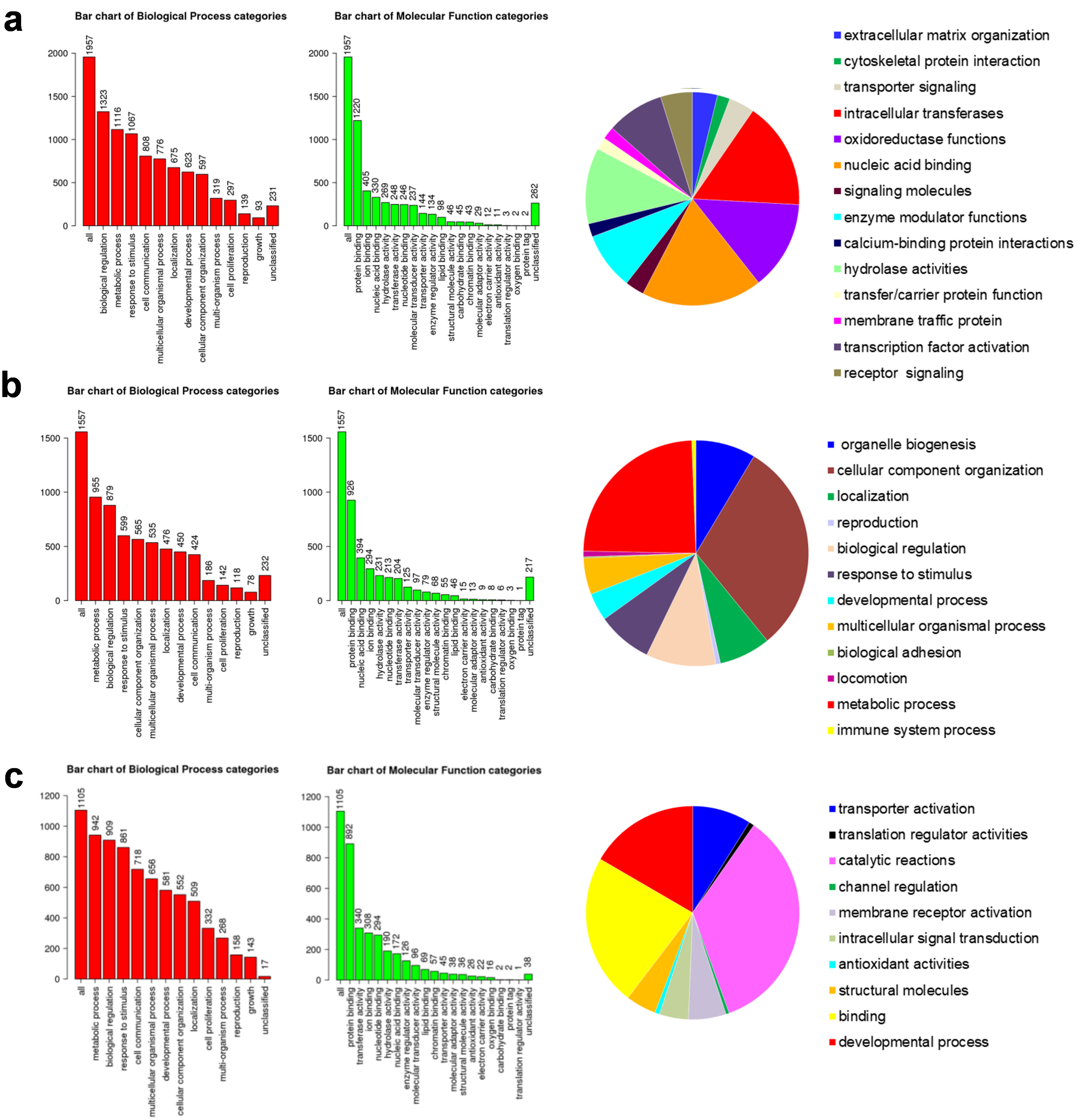

a
Bar chart of Biological Process categories
all 1957
biological regulation 1323
metabolic process 1116
response to stimulus 1067
cell communication 808
multicellular organismal process 776
localization 675
developmental process 623
cellular component organization 597
multi-organism process 319
cell proliferation 297
reproduction 139
growth 93
unclassified 231
Bar chart of Molecular Function categories
all 1957
protein binding 1220
ion binding 405
nucleic acid binding 330
hydrolase activity 269
transferase activity 248
nucleotide binding 246
molecular transducer activity 237
transporter activity 144
enzyme regulator activity 134
lipid binding 98
structural molecule activity 46
carbohydrate binding 45
chromatin binding 43
molecular adaptor activity 29
electron carrier activity 12
antioxidant activity 11
translation regulator activity 3
oxygen binding 2
protein tag 2
unclassified 262
extracellular matrix organization
cytoskeletal protein interaction
transporter signaling
intracellular transferases
oxidoreductase functions
nucleic acid binding
signaling molecules
enzyme modulator functions
calcium-binding protein interactions
hydrolase activities
transfer/carrier protein function
membrane traffic protein
transcription factor activation
receptor signaling
b
Bar chart of Biological Process categories
all 1557
metabolic process 955
biological regulation 879
response to stimulus 599
cellular component organization 565
multicellular organismal process 535
localization 476
developmental process 450
cell communication 424
multi-organism process 186
cell proliferation 142
reproduction 118
growth 78
unclassified 232
Bar chart of Molecular Function categories
all 1557
protein binding 926
nucleic acid binding 394
ion binding 294
hydrolase activity 231
nucleotide binding 213
transferase activity 204
transporter activity 125
molecular transducer activity 97
enzyme regulator activity 79
structural molecule activity 68
chromatin binding 55
lipid binding 46
electron carrier activity 15
molecular adaptor activity 13
antioxidant activity 9
carbohydrate binding 8
translation regulator activity 6
oxygen binding 3
protein tag 1
unclassified 217
organelle biogenesis
cellular component organization
localization
reproduction
biological regulation
response to stimulus
developmental process
multicellular organismal process
biological adhesion
locomotion
metabolic process
immune system process
c
Bar chart of Biological Process categories
all 1105
metabolic process 942
biological regulation 909
response to stimulus 861
cell communication 718
multicellular organismal process 656
developmental process 581
cellular component organization 552
localization 509
cell proliferation 332
multi-organism process 268
reproduction 158
growth 143
unclassified 17
Bar chart of Molecular Function categories
all 1105
protein binding 892
transferase activity 340
ion binding 308
nucleotide binding 294
hydrolase activity 190
nucleic acid binding 172
enzyme regulator activity 126
molecular transducer activity 96
lipid binding 69
chromatin binding 57
transporter activity 45
molecular adaptor activity 38
structural molecule activity 36
antioxidant activity 28
electron carrier activity 22
oxygen binding 16
carbohydrate binding 2
protein tag 2
translation regulator activity 1
unclassified 38
transporter activation
translation regulator activities
catalytic reactions
channel regulation
membrane receptor activation
intracellular signal transduction
antioxidant activities
structural molecules
binding
developmental process

Figure S7

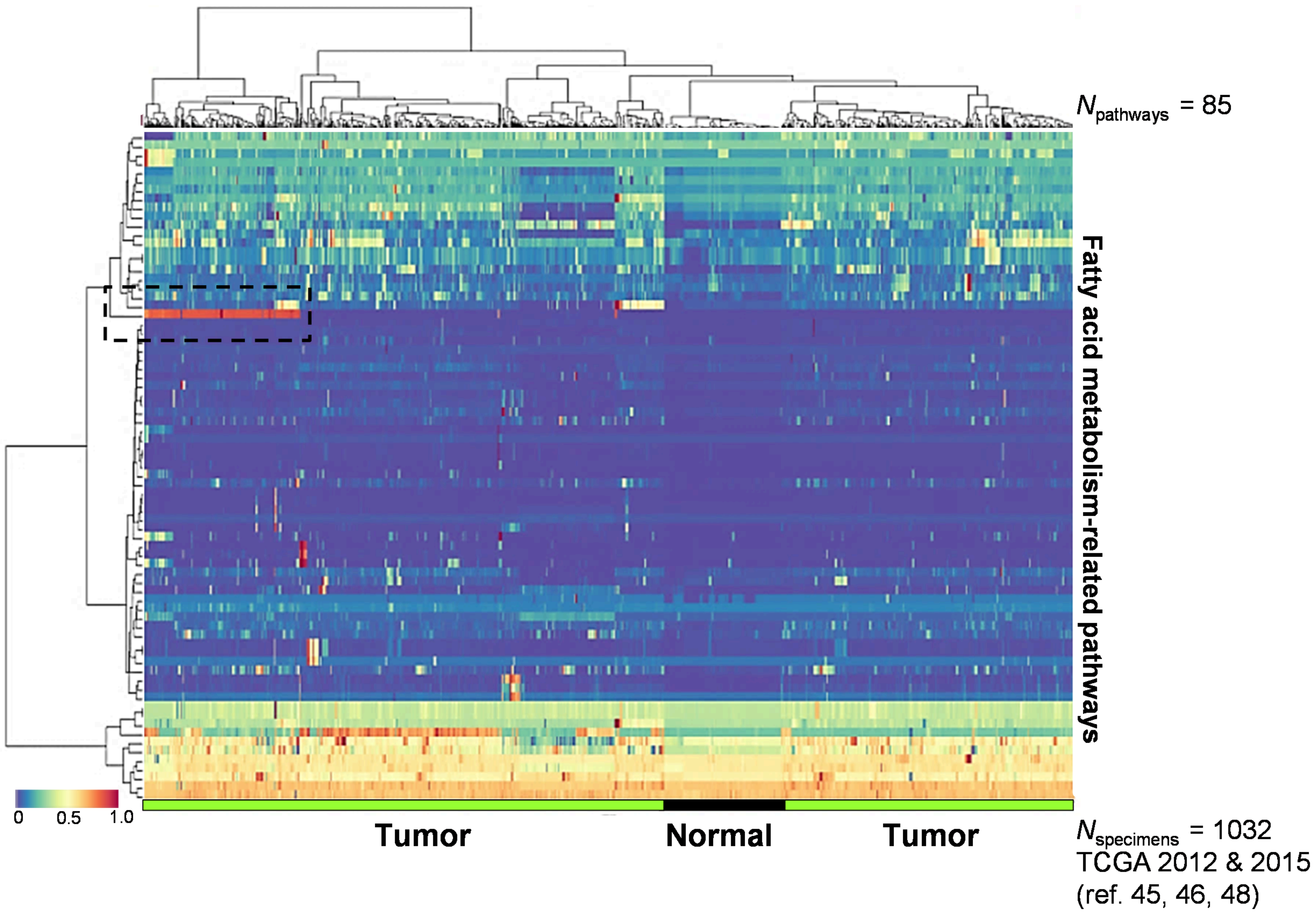

$N_{pathways}$ = 85
Fatty acid metabolism-related pathways
0
0.5
1.0
Tumor
Normal
Tumor
$N_{specimens}$ = 1032
TCGA 2012 & 2015
(ref. 45, 46, 48)

Figure S8

a

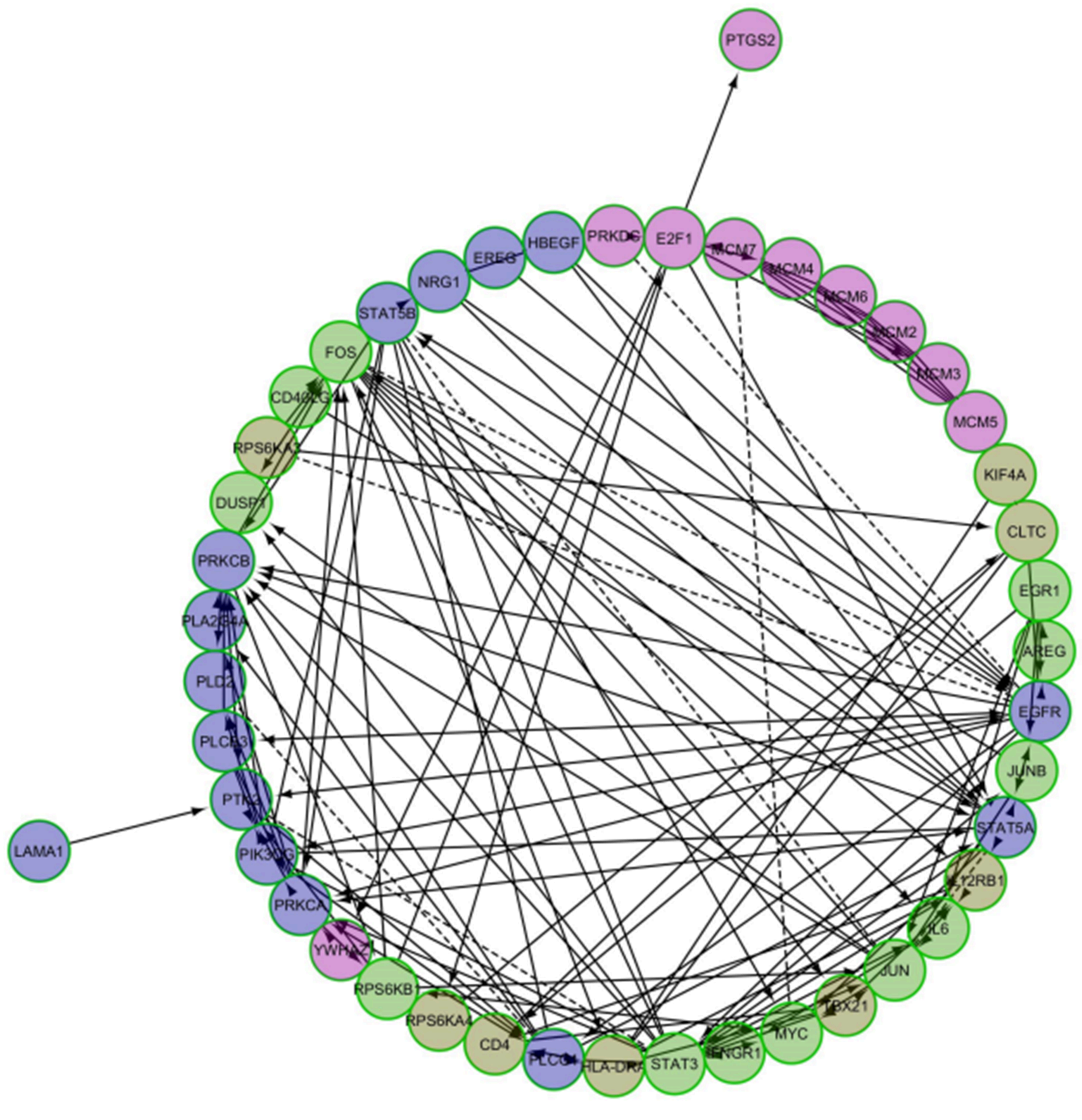

HER2+

Top 10 GO processes	Target
G1/S transition of mitotic cell cycle	STAT5B
MAPK cascade	AREG
mitotic cell cycle	IFNGR1
cell activation	LAMA1
regulation of protein phosphorylation	NRG1
positive regulation of protein phosphorylation	MCM2
immune system process	EREG
leukocyte differentiation	MAP3K5
regulation of immune system process	S1PR3
positive regulation of immune system process	EGR1

b

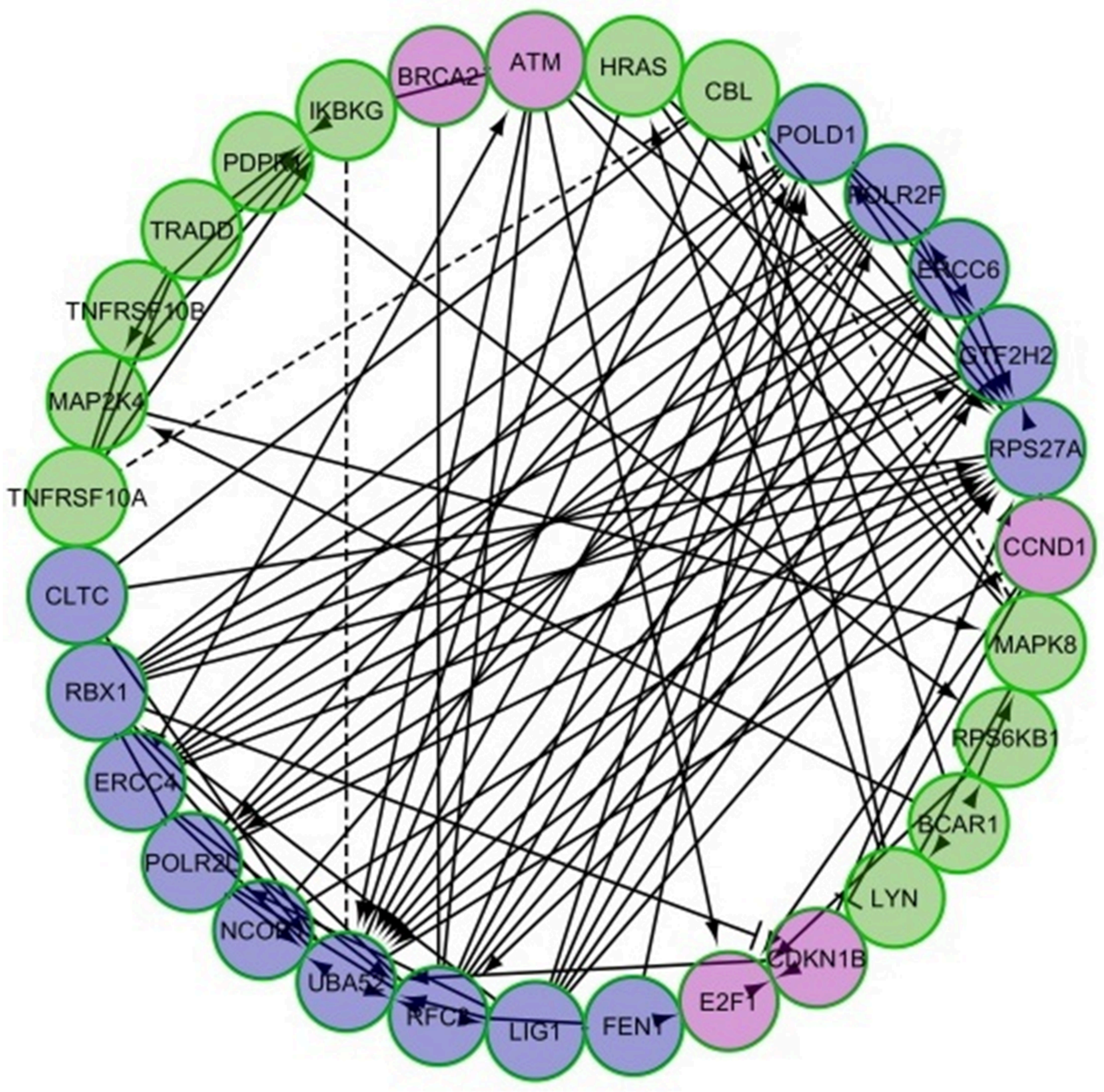

TPBC or ER+/PR+/HER2–

Top 10 GO processes	Target
establishment of planar polarity	GTF2H2B
cellular response to DNA damage stimulus	FEN1
mitotic cell cycle	MAPK8
transcription-coupled nucleotide-excision repair	TNFRSF10B
regulation of protein phosphorylation	GTF2H2
generation of precursor metabolites and energy	CDKN1B
oxidative phosphorylation	BRCA2
mitochondrial electron transport, NADH to ubiquinone	CASP7
purine nucleotide metabolic process	KAT2B
protein complex assembly	ERCC6

c

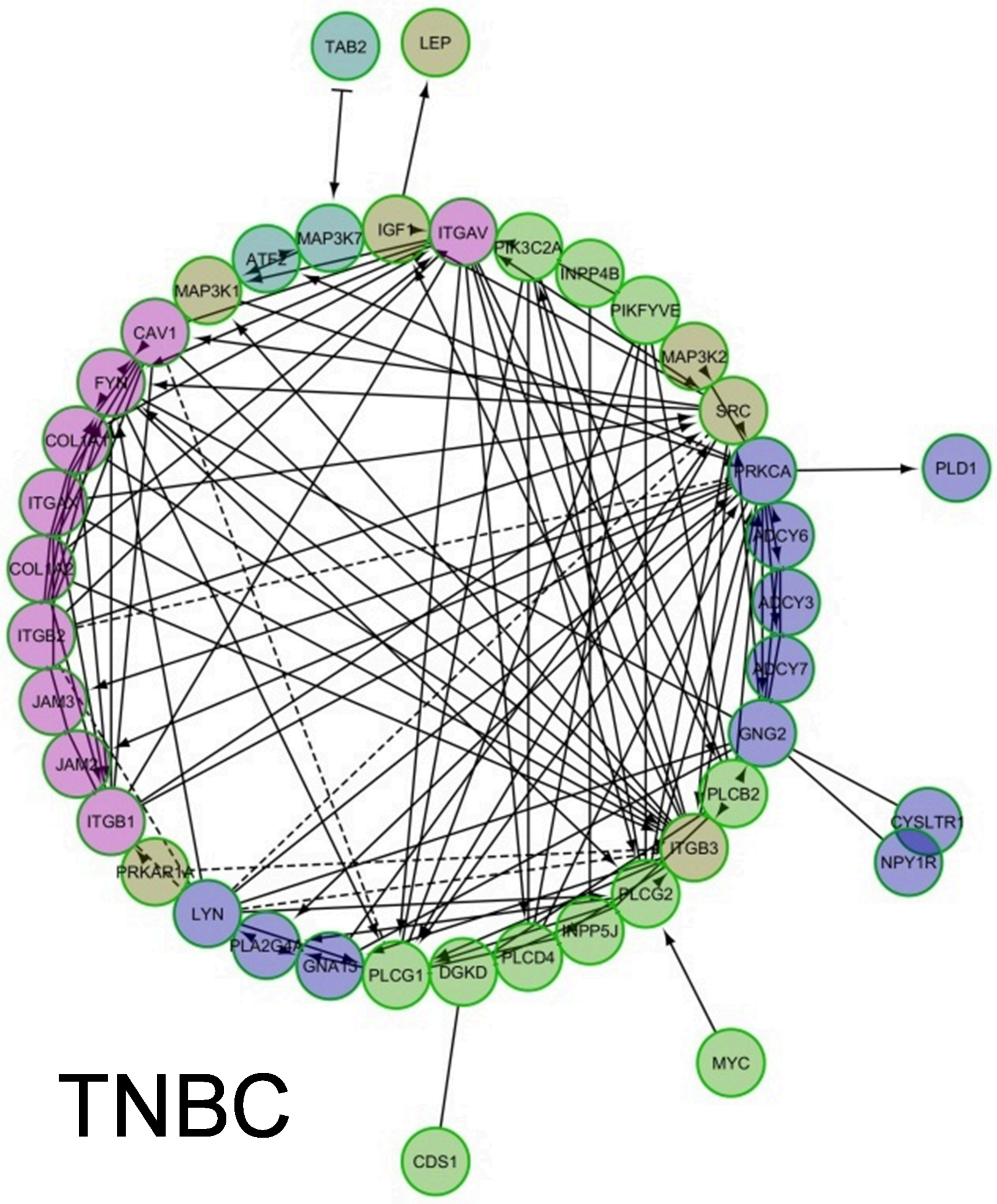

TNBC

Top 10 GO processes	Target
angiogenesis	GSTM4
blood vessel development	SRC
cell activation	ITGAV
cellular lipid metabolic process	LPCAT3
positive regulation of protein phosphorylation	CYSLTR1
vasculature development	DGKG
system process	ITGAM
circulatory system process	ITPKA
phosphorus metabolic process	SLC2A1
angiogenesis	GSTM1
